# Algorithmic Guardrails for Echocardiography AI: Deep Feature Representations for Error Slicing and Selective LVEF Estimation

**DOI:** 10.1016/j.jacadv.2026.103333

**Published:** 2026-10-13

**Authors:** Krish Kabra, Sadeer Al-Kindi, Guha Balakrishnan

**Affiliations:** a Department of Electrical and Computer Engineering, Rice University, Houston, Texas, USA; b Center for Cardiovascular Computational and Precision Health, Department of Cardiology, DeBakey Heart and Vascular Center, Houston Methodist, Houston, Texas, USA

**Keywords:** deep learning, echocardiography, left ventricular ejection fraction, risk control, trustworthy AI

## Abstract

**Background:**

Automated echocardiography analysis using artificial intelligence (AI) currently lacks effective guardrails or oversight mechanisms to ensure its safe and reliable deployment.

**Objectives:**

This study investigated whether deep feature representations can identify echocardiograms at risk of misinterpretation by echocardiography AI-based left ventricular ejection fraction (LVEF) estimation models.

**Methods:**

We retrospectively validated 2 state-of-the-art models for LVEF estimation, EchoNet (single-task model) and PanEcho (multitask foundation model), on 4,010 apical-4-chamber videos from 1,353 studies (n = 1,208) with biplane LVEF measurements in the MIMIC-IV-ECHO database (2017-2019). We extracted 4 deep feature representations from a view classifier, a left ventricle segmentation model, and the 2 validated LVEF estimation models. These features were evaluated for their ability to identify systematic misinterpretations (error slicing) and automatically exclude AI estimates for high-risk inputs (selective estimation).

**Results:**

While PanEcho performed better relative to EchoNet (EchoNet minus PanEcho [Δ], Δmean absolute error: 0.31 [0.07, 0.55]; ΔR^2^: −0.07 [−0.11, −0.03]), both models systematically overestimated LVEF at lower values (proportional bias slope, EchoNet: −0.33 [−0.39, −0.28], *P* < 0.001, PanEcho: −0.31 [−0.36, −0.26], *P* < 0.001). All investigated deep feature representations identified error slices for both models. Shared error slices between the models revealed 2 common failure modes: contrast-enhanced echocardiograms and poor-quality apical-4-chamber views. Selective estimation using intermediate features from EchoNet achieved the best performance across both estimation models (area under risk-coverage curve, EchoNet: 5.04 [4.45, 5.66]; PanEcho: 5.21 [4.54, 5.92]).

**Conclusions:**

Deep feature representations can be leveraged to identify and automatically flag misinterpretation-prone echocardiograms, enabling promising guardrail mechanisms for the reliable deployment of echocardiography AI.

Echocardiography is an essential diagnostic imaging modality for diagnosing and monitoring various cardiovascular conditions. Over the past few years, artificial intelligence (AI) algorithms driven by deep learning (DL) have enabled the automatic interpretation of echocardiograms. Traditional DL algorithms trained to estimate one or a few metrics from echocardiograms, such as left ventricular ejection fraction (LVEF)^1^, ^2^, ^3^, ^4^, ^5^, ^6^ and wall thickness,^7^^,^^8^ are now achieving Food and Drug Administration clearance and clinical viability,^9^^,^^10^ while recent DL “foundation models” trained to generate a range of metrics or text reports offer comprehensive interpretations.^11^, ^12^, ^13^

While these algorithms promise to significantly reduce analysis time and burden for sonographers and cardiologists, they also inherit well-known generalization vulnerabilities of DL models, leading to potential performance gaps across patient subgroups and hospital sites.^14^, ^15^, ^16^, ^17^, ^18^, ^19^, ^20^ AI performance can vary widely in real clinical applications due to data heterogeneity. This issue is particularly evident in echocardiography, where data are heterogeneous and noisy due to variations in ultrasound devices, acquisition protocols, sonographer expertise, and patient demographics. Consequently, deployed algorithms may encounter inputs that deviate substantially from their training distribution, including poor-quality acquisitions, nonstandard views, and under-represented patient populations, which can result in unreliable predictions and misinterpretations. While completely eliminating these generalization gaps may not be feasible, methods to determine when an algorithm's predictions are trustworthy can mitigate the risks of downstream misdiagnoses, harm to subpopulations, and erosion of clinician and patient trust.^21^, ^22^, ^23^, ^24^ Therefore, algorithmic guardrails are urgently needed to ensure we can trust the outputs of echocardiography AI.

**Central Illustration:**
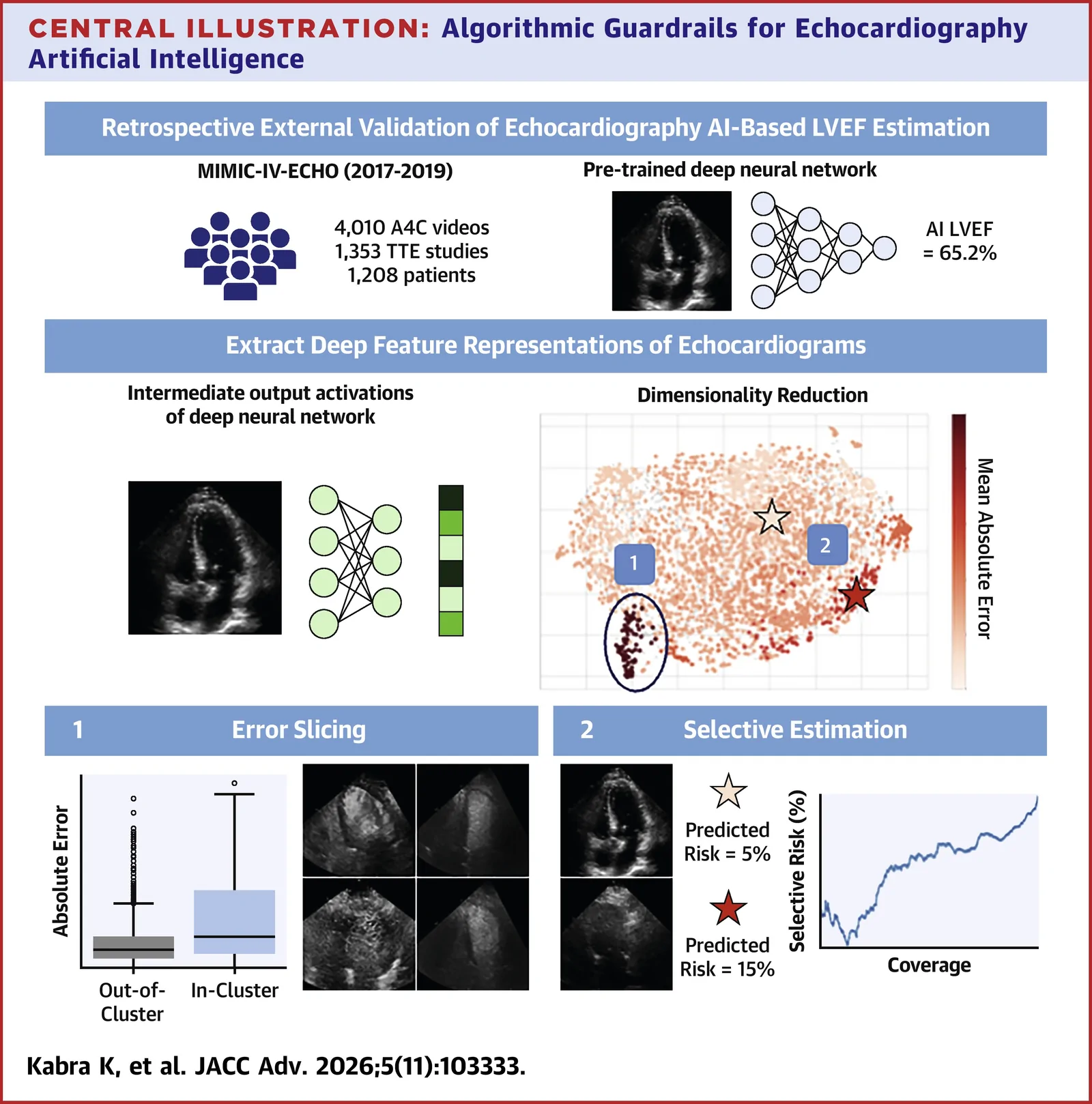
Algorithmic Guardrails for Echocardiography Artificial Intelligence Echocardiograms captured in clinical practice are heterogeneous and noisy, leading to highly variable performance of echocardiography artificial intelligence. To mitigate this, we investigated using feature representations extracted from intermediate-layer activations of deep neural networks to identify echocardiograms prone to artificial intelligence misinterpretation during left ventricular ejection fraction estimation. Features were extracted from 4 complementary networks: a view classifier, a left ventricle segmentation model, and 2 left ventricular ejection fraction estimation models, EchoNet and PanEcho. We demonstrated that these features can be used for: 1) error slicing, identifying groups of echocardiograms sharing characteristics that systematically cause artificial intelligence misinterpretations; and 2) selective estimation, where an estimated misinterpretation-risk score from a trained model determines whether to retain or reject an echocardiogram for artificial intelligence–based estimation. Adjusting the risk threshold produces a trade-off between coverage and estimation accuracy, enabling clinicians to tailor the level of automated oversight to their tolerance for risk. A4C = apical-4-chamber; TTE = transthoracic echocardiography; other abbreviations as in Figure 1.

Traditionally, these guardrails have been enabled by image quality estimation techniques,^25^, ^26^, ^27^ which have 2 shortcomings. First, they require expert annotations of image quality, which are not readily available in clinical databases or in standard echocardiography reports. Second, they assume that prediction errors stem exclusively from perceptible image quality attributes, ignoring complex, imperceptible data patterns.

To address these limitations, we propose an approach to identify echocardiograms at high risk of misinterpretation by a given echocardiography AI-based LVEF estimation model, relying only on readily available historical LVEF measurements. The proposed approach is predicated on the hypothesis that AI algorithms exhibit systematic error patterns within semantically or perceptually coherent clusters of data, known as *error* slices.^28^, ^29^, ^30^, ^31^ Crucially, deep feature representations derived from neural network intermediate activations can capture the perceptual and semantic properties that distinguish error-prone inputs, without requiring manual specification of each failure mode. In doing so, these deep features offer a robust way to identify which data points the AI model performs poorly on. We leveraged feature representations from 4 distinct networks: an echocardiogram view classifier, a left ventricle (LV) segmentation model, and the 2 LVEF estimation models evaluated in this study, EchoNet^1^ and PanEcho.^11^ Using these representations, we first investigated their ability to identify error slices of both models. We then assessed whether these deep feature representations could be utilized for selective estimation^22^^,^^32^, ^33^, ^34^, ^35^ of LVEF, that is, automatically deciding which echocardiograms will yield accurate AI-based LVEF estimates and abstaining from the rest (Central Illustration).

## Methods

### Data

We curated data for the study from the publicly available MIMIC-IV-ECHO database,^36^ which contains echocardiograms and structured measurements from transthoracic echocardiography studies collected across Beth Israel Deaconess Medical Center from patients admitted between 2017 and 2019. We identified 1,521 studies from 1,344 patients with LVEF measurements by Simpson’s biplane method. Following established protocols,^11^^,^^37^ we preprocessed all echocardiogram videos and classified them into 23 fine-grained views using a validated view classifier.^4^ We selected only videos from the apical-4-chamber (A4C) viewpoint, the primary viewpoint used for LVEF evaluation by echocardiography analysis models, resulting in a total of 4,010 videos from 1,353 studies of 1,208 patients. For full details on LVEF measurement curation, video preprocessing, and patient and study characteristics, please refer to the Supplemental Methods, Supplemental Figure 1, and Supplemental Table 1.

### Echocardiography AI-based LVEF estimation models

We focused our investigations on 2 prominent, open-source DL algorithms for estimating LVEF from A4C videos: EchoNet^1^ and PanEcho.^11^ EchoNet is a spatiotemporal convolutional neural network trained to estimate LVEF using 7,465 A4C videos collected from patients who underwent echocardiography as part of clinical care at Stanford Health Care. PanEcho is a video-based DL model trained to generate 39 echocardiographic reporting outputs, including LVEF, using 999,727 videos from 25,130 studies collected from patients who underwent echocardiography as part of clinical care at Yale New Haven Health System hospitals. Because both algorithms estimate LVEF on a “beat-to-beat” basis, we obtained a single measurement for each record by averaging estimates across all sampled clips from each video. MIMIC-IV-ECHO was not part of the training data sets of either algorithm. We provide a comprehensive overview of the inference pipeline in Supplemental Methods.

### Deep feature representations of echocardiograms

Intermediate layer activations in deep neural networks effectively encode rich perceptual and semantic image concepts.^38^, ^39^, ^40^, ^41^, ^42^, ^43^ We extracted intermediate outputs from 4 distinct neural networks that capture complementary aspects of echocardiographic data: an echocardiogram view classifier^4^ (the same network used for A4C view classification during data curation), an LV segmentation model,^1^ and the 2 LVEF estimation models investigated in this study, EchoNet and PanEcho. We then investigated which of these features correlate with echocardiography AI-based LVEF estimation errors.

Representations from the view classifier encode global structural features of standard echocardiographic views, while the LV segmentation model captures spatial and textural details of LV morphology. Together, these complementary models provide domain-specific features that can identify common sources of LVEF estimation errors, such as ambiguous imaging planes or poor endocardial visualization. In contrast, deep feature representations from EchoNet and PanEcho, which we refer to as EchoNet-Feats and PanEcho-Feats, reflect the internal concepts learned by the models for estimating LVEF. By including these representations, we assessed whether a LVEF estimation model's learned features can identify its own misinterpretations or those of an alternate estimation model.

Following common practices in existing perceptual vision studies,^43^, ^44^, ^45^ we extracted output activations from the final pooling layers of convolutional neural network-based models (view classifier, LV segmentation model, and EchoNet) and the final “CLS” tokens of vision transformer-based models (PanEcho). Because the view classifier and LV segmentation model operate on single-image inputs, we derived video-level feature representations by computing both the mean and the top 3 principal components of the output activations across all frames (the principal components were included to preserve temporal variation in frame-level features that mean pooling alone would obscure). Because EchoNet and PanEcho operate on video clips, we derived video-level feature representations by computing the mean of the output activations across all sampled clips per video.

### Error slicing

To investigate whether the proposed deep feature representations can identify misinterpretation-prone echocardiograms, we first used them to perform *error slicing*, the task of clustering data points into groups sharing perceptually salient characteristics and similar downstream errors from a prediction algorithm.^28^, ^29^, ^30^, ^31^ Error slicing was performed independently for each combination of feature representation and LVEF estimation model.

Given a deep feature representation, we first embedded all echocardiograms in the data set into the feature space and identified clusters using a combination of the Uniform Manifold Approximation and Projection^46^ (UMAP) dimensionality reduction algorithm and the Hierarchical Density-Based Spatial Clustering of Applications with Noise^47^ clustering algorithm. Because these methods are sensitive to both random initialization and parameter settings, we obtained stable clusters across multiple seeds and parameter settings by running a consensus clustering algorithm based on evidence accumulation.^48^ Complete details of the clustering pipeline and hyperparameters are provided in the Supplemental Methods, along with a clustering stability analysis (Supplemental Table 2).

After identifying the clusters, we then identified the error slices by comparing the LVEF estimation model’s performance on echocardiograms contained in each cluster with that outside it. For each cluster, we estimated the differences in absolute error means between in-cluster and out-of-cluster records. Clusters with differences significantly greater than zero were defined as error slices (see Statistical Analysis for statistical hypothesis testing details).

### Selective estimation

We next evaluated whether deep feature representations could enable *selective estimation*, a process that automatically abstains from generating AI-based LVEF estimates for echocardiograms at high risk of misinterpretation.^22^^,^^32^, ^33^, ^34^, ^35^ As with error slicing, selective estimation was performed independently for each combination of feature representation and LVEF estimation model.

We adapted the proposed error-slicing approach to a supervised framework. Given a deep feature representation and an LVEF estimation model, we trained a model to predict the misinterpretation risk of an input echocardiogram, defined as the absolute error between the AI-based estimate and the reported biplane LVEF. We partitioned the MIMIC-IV-ECHO data set at the patient level into training, validation, and test subsets in a 70:10:20 ratio. For each feature representation, all echocardiograms were embedded into the feature space, and a UMAP dimensionality reduction was fit on the training-set embeddings. A k-nearest neighbors regression model was then trained to predict misinterpretation risk from each echocardiogram’s dimensionally reduced embedding. The UMAP and k-nearest neighbors hyperparameters were jointly selected by grid search to maximize agreement between predicted and observed misinterpretation risk on the validation set (see Supplemental Table 3 for search configurations).

After training the misinterpretation risk estimator, selective estimation was performed by setting a threshold that prevented AI-LVEF estimates from being generated for echocardiograms with a predicted risk that exceeded it. Varying this threshold produced a coverage-performance trade-off, where stricter (smaller-valued) thresholds ideally improved overall LVEF estimation performance at the cost of more abstentions. An effective selective estimator should achieve both high performance and minimal abstentions.

### Statistical analysis

Because the data have a nested structure, videos are nested in studies, and studies are nested in patients, the analyses accounted for this nonindependence. All 95% CIs for performance metrics were computed using a patient-level percentile bootstrap, with patients resampled with replacement over 10,000 iterations. Similarly, differences in metrics between methods were computed using the same bootstrap applied in a paired fashion, that is, resampling patients once per iteration and recomputing the paired difference, and were considered significant when their 95% bootstrap CI excluded zero. All ordinary least squares (OLS) and logistic regressions used cluster-robust standard errors^49^ with patients as the clusters, and coefficients were considered significant with a 2-sided test at α = 0.05, unless specified otherwise. The analytic unit (ie, videos, studies, or patients) for each analysis is specified below. All statistical analyses were performed in Python 3.12 using NumPy v2.2.0, statsmodels v0.14.4, and scikit-learn v1.8.0.

#### LVEF estimation model evaluation

The performance of LVEF estimation models was assessed at the video level, evaluating agreement between the AI-based LVEF estimates and the reference biplane LVEF. Continuous agreement was quantified using the mean absolute error (MAE), root mean squared error, Pearson correlation coefficient (R), Lin's concordance correlation coefficient, and coefficient of determination (R^2^). Additionally, agreement across clinically meaningful LVEF ranges was quantified by categorizing LVEF into <40%, 40% to 50%, and >50%, which correspond to the 2022 American Heart Association/American College of Cardiology/Heart Failure Society of America heart failure classifications,^50^ and computing the linearly weighted kappa (κ_w_).^51^ Agreement was further characterized using Bland-Altman plots. Because the conventional limits-of-agreement assume constant bias and variance across the measurement range, these assumptions were explicitly evaluated. Proportional bias was assessed by OLS regression of the intermethod differences on the intermethod means, and magnitude-dependent variability (ie, heteroskedasticity) was assessed by OLS regression of the absolute residuals from the proportional bias regression on the intermethod means. When evidence of proportional bias and/or magnitude-dependent variability was present, regression-based Bland-Altman limits of agreement were used.

#### Error slicing

The difference in absolute error means between in-cluster and out-of-cluster records was estimated at the video level by OLS regression of each record's absolute error on a binary indicator of cluster membership (the coefficient equals the mean difference). Because error slices are clusters with elevated error (ie, a positive mean difference), significance was assessed with a one-sided test at α = 0.05. To characterize the resulting error slices, we summarized LVEF agreement among in-slice videos using the continuous agreement metrics and Bland-Altman analyses described above. Additionally, we compared patient- and study-level characteristics (age, sex, race, obesity [body mass index ≥30 kg/m^2^], ultrasound device, and reference LVEF) between in-slice and out-of-slice studies by regressing each characteristic on a binary slice-membership indicator (OLS for continuous characteristics and logistic for categorical characteristics, with multiclass categories assessed one-versus-all). A study was classified as in-slice if at least one of its videos was in-slice.

#### Selective estimation

Selective estimation performance was assessed at the study level on the held-out test set. For studies containing multiple A4C videos, the video with the lowest predicted risk score was selected as the representative from which the AI-based LVEF estimate was generated, and a study was retained (ie, not abstained from) only when its representative risk score fell below a specified threshold. We quantified selective estimation performance using the area under the risk-coverage curve^33^^,^^35^ (AURC), where coverage is the proportion of retained studies and selective risk is the MAE across them, the selective risk at fixed coverage levels (R@C) of 20%, 50%, and 90%, and the coverage at selective risk (C@R) of 5 LVEF points. To further assess whether selective estimation improved performance relative to standard AI deployment, we measured the difference between the AURC and the MAE of a no-selection baseline, in which study-level AI-based LVEF estimates were taken as the mean across all A4C videos in the study.

### Data ethics and software availability

Data from the MIMIC-IV-ECHO database are anonymized and made publicly available by PhysioNet.^52^ The original data were approved by the Institutional Review Boards of Beth Israel Deaconess Medical Center (Boston, MA) and the Massachusetts Institute of Technology (Cambridge, MA). All authors who accessed the data were credentialed PhysioNet users and complied with the applicable data use agreement. We make all code available at https://github.com/krishk97/echo-ai-guardrails.

## Results

### LVEF estimation performance on MIMIC-IV-ECHO

Table 1 and Figure 1 show the LVEF estimation performance of EchoNet and PanEcho on the curated MIMIC-IV-ECHO data. Both models showed moderate overall agreement with the reference biplane LVEF (EchoNet and PanEcho, MAE: 6.62 [95% CI: 6.29, 6.96] and 6.32 [6.03, 6.61]; R^2^: 0.59 [0.54, 0.64] and 0.66 [0.61, 0.70]; κ_w_: 0.68 [0.64, 0.72] and 0.74 [0.69, 0.78]). Across all considered evaluation metrics, LVEF estimates by PanEcho agreed more closely with the reference than EchoNet (EchoNet minus PanEcho [Δ], ΔMAE: 0.31 [0.07, 0.55]; ΔR^2^: −0.07 [−0.11, −0.03]; Δκ_w_: −0.06 [−0.09, −0.02]).

**Table 1:** LVEF Estimation Evaluation Metrics on MIMIC-IV-ECHO

Model	EchoNet	PanEcho	Δ
MAE, %	6.62 (6.29, 6.96)	6.32 (6.03, 6.61)	0.31 (0.07, 0.55)
RMSE, %	8.87 (8.30, 9.45)	8.07 (7.74, 8.41)	0.79 (0.35, 1.26)
R	0.77 (0.74, 0.80)	0.82 (0.79, 0.85)	−0.05 (−0.08, −0.03)
CCC	0.74 (0.70, 0.77)	0.79 (0.75, 0.81)	−0.05 (−0.08, −0.02)
R^2^	0.59 (0.54, 0.64)	0.66 (0.61, 0.70)	−0.07 (−0.11, −0.03)
κ_w_	0.68 (0.64, 0.72)	0.74 (0.69, 0.78)	−0.06 (−0.09, −0.02)

Agreement metrics between AI-based and reference biplane LVEF estimates for EchoNet and PanEcho, and their differences (EchoNet minus PanEcho). Point values are computed across all videos curated in the MIMIC-IV-ECHO data set, and sets are 95% CIs computed using a patient-level percentile bootstrap, with patients resampled with replacement over 10,000 iterations.

AI = artificial intelligence; CCC = Lin’s concordance correlation coefficient; κ_w_ = linear weighted kappa; MAE = mean absolute error; R = Pearson correlation coefficient; R^2^ = coefficient of determination; RMSE = root mean-squared error.

**Figure 1:**
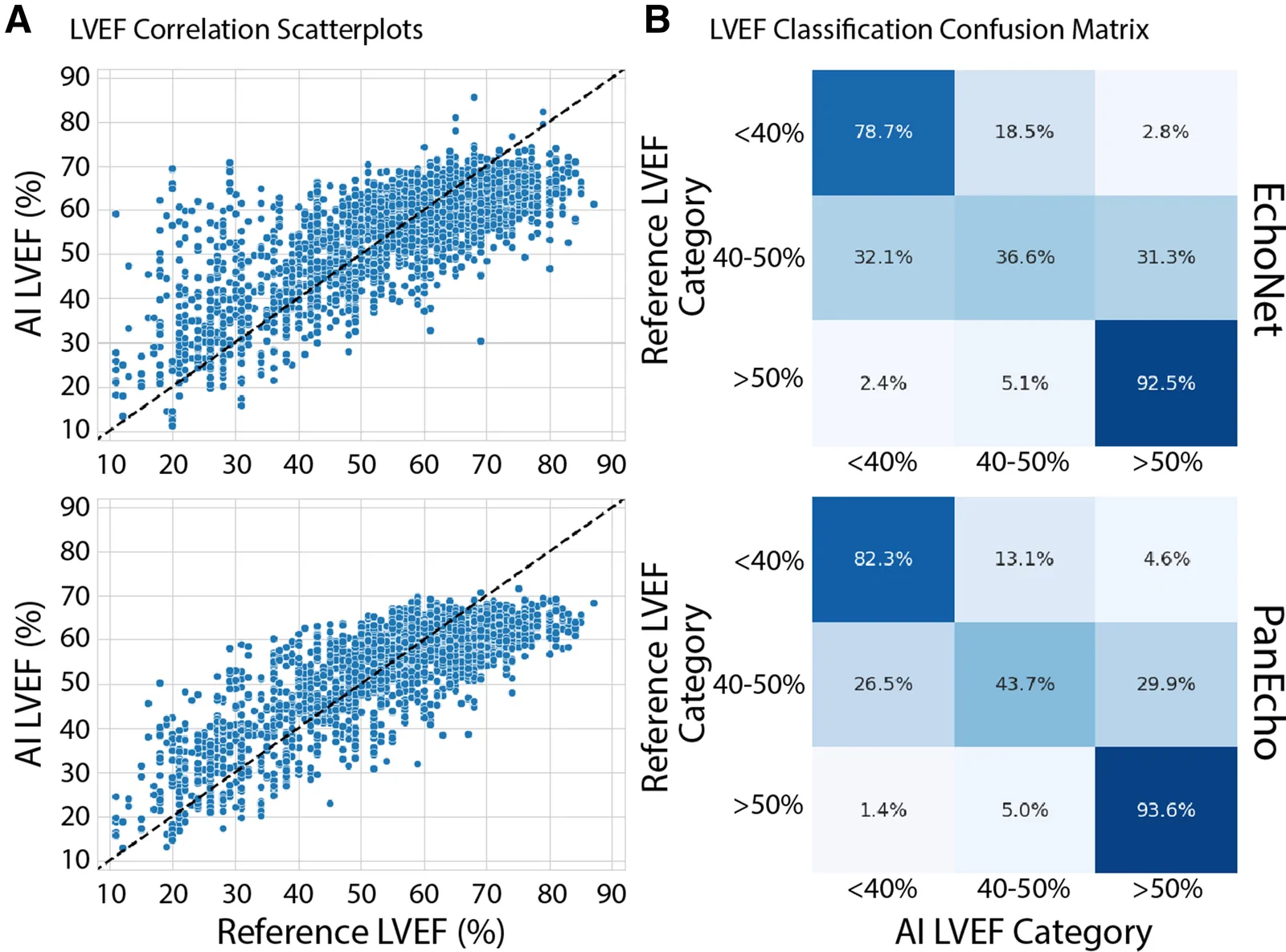
Performance Evaluation of Left Ventricular Ejection Fraction Estimation Models on MIMIC-IV-ECHO Performance evaluation plots for left ventricular ejection fraction estimates from EchoNet (top row) and PanEcho (bottom row) on the curated MIMIC-IV-ECHO data set. (A) Reference vs artificial intelligence–generated left ventricular ejection fraction correlation scatterplots, with the dashed line representing perfect correlation (y = x). (B) Confusion matrices for left ventricular ejection fraction classification across critical ranges used for distinguishing types of heart failure. AI = artificial intelligence; LVEF = left ventricular ejection fraction.

Despite this difference in overall agreement, both models exhibited significant proportional bias and magnitude-dependent variability (Figure 2, Table 2). Each tended to overestimate LVEF at lower values and underestimate it at higher values (EchoNet and PanEcho, proportional bias, intercept: 18.47 [15.29, 21.66], *P* < 0.001 and 16.16 [13.37, 18.95], *P* < 0.001; slope: −0.33 [−0.39, −0.28], *P* < 0.001 and −0.31 [−0.36, −0.26], *P* < 0.001). Furthermore, both models showed greater variability in intermethod differences at lower reference LVEF (EchoNet and PanEcho, magnitude-dependent variability slope: −0.12 [−0.15, −0.09], *P* < 0.001 and −0.09 [−0.11, −0.06], *P* < 0.001). Stratifying the absolute LVEF error by category (Figure 2B) revealed a consistent pattern: the median and IQR for errors were largest in the <40% category and smallest in the >50% category.

**Figure 2:**
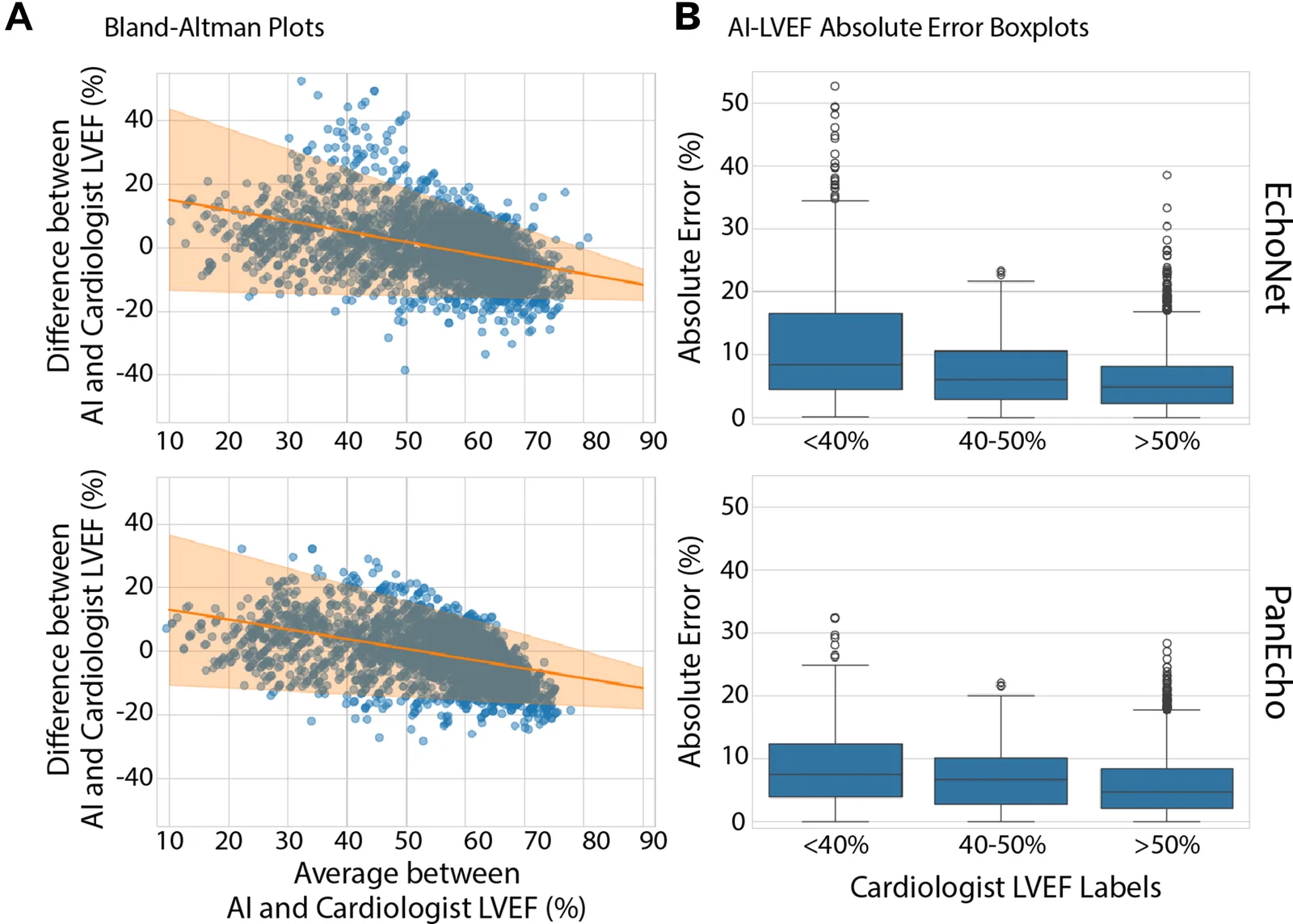
Proportional Bias and Magnitude-Dependent Variability of Left Ventricular Ejection Fraction Estimation Models in MIMIC-IV-ECHO Plots showing proportional bias and magnitude-dependent variability (ie, heteroskedasticity) in left ventricular ejection fraction estimation from EchoNet (top row) and PanEcho (bottom row) on the curated MIMIC-IV-ECHO data set. (A) Bland-Altman plots: the solid line is the proportional-bias regression (difference regressed on average) and the shaded band is the 95% limits of agreement from the magnitude-dependent variability regression. (B) Box-plots of absolute left ventricular ejection fraction estimation errors stratified by critical ranges. Abbreviations as in Figure 1.

**Table 2:** Proportional Bias and Magnitude-Dependent Variability Coefficients for LVEF Estimation Models on MIMIC-IV-ECHO

	EchoNet		PanEcho	
	Coefficient (95% CI)	*P* Value	Coefficient (95% CI)	*P* Value
Proportional bias: intercept, %	18.47 (15.29, 21.66)	<0.001	16.16 (13.37, 18.95)	<0.001
Proportional bias: slope	−0.33 (−0.39, −0.28)	<0.001	−0.31 (−0.36, −0.26)	<0.001
Magnitude-dependent variability: intercept, %	12.83 (11.18, 14.48)	<0.001	10.49 (9.05, 11.93)	<0.001
Magnitude-dependent variability: slope	−0.12 (−0.15, −0.09)	<0.001	−0.09 (−0.11, −0.06)	<0.001

Coefficients for proportional bias and magnitude-dependent variability OLS regression analyses of EchoNet and PanEcho on the curated MIMIC-IV-ECHO data set (proportional bias: intermethod differences on the intermethod means; magnitude-dependent variability: absolute residuals from the proportional bias regression on the intermethod means; intermethod refers to AI-based vs reference LVEF estimates). Regressions used cluster-robust standard errors with patients as the clusters.

LVEF = left ventricular ejection fraction; OLS = ordinary least squares; other abbreviation as in Table 1.

### Error slicing results

Identified error slices for each deep feature representation and LVEF estimation model are visualized in Figure 3 and Supplemental Figure 2 (Supplemental Table 4 summarizes estimated MAE differences between in-slice and out-of-slice videos for all identified error slices). Each feature space identified error slices for both models, with several slices shared between the 2 models (ie, groups of echocardiograms both models exhibited significantly elevated MAE on). A visual review of the videos in the shared error slices reveals 2 recurring characteristics associated with LVEF estimation errors: contrast-enhanced echocardiograms (Figures 3Ai and 3Ci) and poor-quality, ambiguous, or potentially mislabeled A4C views (Figures 3Aii and 3Dii). Another error slice, specific to EchoNet and with a discernible visual characteristic, comprised of zoomed-in A4C views of the LV (Figure 3Di).

**Figure 3:**
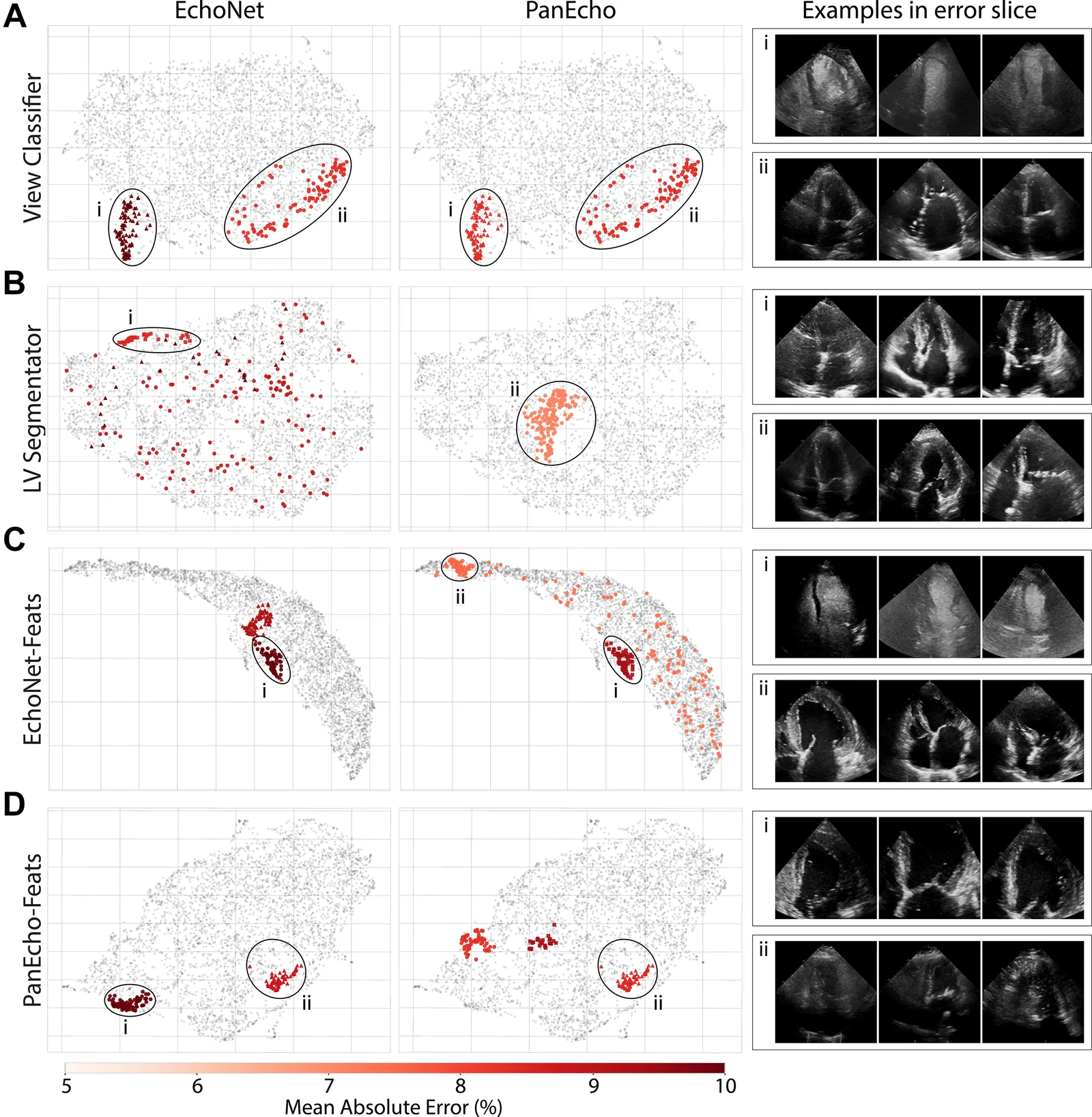
Error Slices Identified by Deep Feature Representations of Echocardiograms Each row corresponds to a deep feature representation used for error slicing: (A) view classifier, (B) left ventricle segmentator, (C) EchoNet-Feats, (D) PanEcho-Feats. In columns 1 to 2, each point is an echocardiogram positioned by a 2-dimensional UMAP projection of that representation. Both columns share this embedding but highlight the error slices specific to EchoNet (column 1) and PanEcho (column 2). Colored points belong to an error slice (gray points do not), with marker shapes distinguishing slices and colors encoding each slice's mean absolute error (clipped to 5% to 10%). Slices were identified by clustering in higher dimensions and may not appear compact in this 2-dimensional projection. Column 3 shows example echocardiograms from the circled slices labeled (i) and (ii). LV = left ventricle; UMAP = uniform manifold approximation and projection.

Table 3 and Figure 4 present LVEF estimation performance for both models across the 2 shared view classifier error slices (Supplemental Tables 5 to 7 and Supplemental Figures 3 to 5 present analogous results for identified error slices using other feature spaces). For videos in the contrast-enhanced error slice, EchoNet showed poor agreement with the reference LVEF (MAE: 14.84 [10.44, 19.34], R: 0.22 [−0.03, 0.49], R^2^: −0.45 [−1.15, 0.05]), with significant proportional bias and magnitude-dependent variability (proportional bias slope: −1.24 [−1.61, −0.86], *P* < 0.001; magnitude-dependent variability slope: −0.40 [−0.59, −0.22], *P* < 0.001). The correlation plot (Figure 4B, top row) indicates that this disagreement stems from EchoNet estimating LVEF within a narrow range of 50% to 70% regardless of the reference value. PanEcho, on the other hand, showed moderate agreement for contrast-enhanced videos (MAE: 8.33 [6.74, 9.91], R: 0.82 [0.72, 0.88], R^2^: 0.61 [0.44, 0.71]), with small proportional bias and insignificant magnitude-dependent variability (proportional bias slope: −0.44 [−0.58, −0.30], *P* < 0.001; magnitude-dependent variability slope: −0.07 [−0.15, 0.00], *P* = 0.056). For the poor-quality, ambiguous, or mislabeled A4C slice, agreement was moderate for both models (EchoNet and PanEcho, MAE: 8.34 [6.83, 9.89] and 8.39 [7.15, 9.65]; R: 0.76 [0.64, 0.84] and 0.80 [0.69, 0.87]; R^2^: 0.56 [0.39, 0.66] and 0.62 [0.43, 0.70]), with modestly elevated in-slice MAE (EchoNet and PanEcho, in-slice minus out-of-slice, ΔMAE: 1.77 [0.27, 3.27], *P* = 0.010 and 2.13 [0.88, 3.38], *P* < 0.001) and significant proportional bias (EchoNet and PanEcho, proportional bias slope: −0.44 [−0.61, −0.26], *P* < 0.001, and −0.41 [−0.58, −0.25], *P* < 0.001).

**Table 3:** LVEF Estimation Evaluation Metrics on Echocardiograms From View Classifier Error Slices

	EchoNet		PanEcho	
	Value (95% CI)	*P* Value	Value (95% CI)	*P* Value
Error slice: contrast-enhanced views (Figure 3Ai)				
MAE, %	14.84 (10.44, 19.34)	-	8.33 (6.74, 9.91)	-
RMSE, %	20.04 (14.42, 24.97)	-	10.41 (8.51, 12.10)	-
R	0.22 (−0.03, 0.49)	-	0.82 (0.72, 0.88)	-
CCC	0.11 (−0.01, 0.31)	-	0.74 (0.64, 0.81)	-
R^2^	−0.45 (−1.15, 0.05)	-	0.61 (0.44, 0.71)	-
Proportional bias: intercept, %	79.15 (55.71, 102.59)	<0.001	25.59 (17.82, 33.35)	<0.001
Proportional bias: slope	−1.24 (−1.61, −0.86)	<0.001	−0.44 (−0.58, −0.30)	<0.001
Magnitude-dependent variability: intercept, %	30.89 (19.85, 41.92)	<0.001	10.14 (5.91, 14.36)	<0.001
Magnitude-dependent variability: slope	−0.40 (−0.59, −0.22)	<0.001	−0.07 (−0.15, 0.00)	0.056
Error slice: poor quality, ambiguous, or mislabeled A4C views (Figure 3Aii)				
MAE, %	8.34 (6.83, 9.89)	-	8.39 (7.15, 9.65)	-
RMSE, %	10.58 (8.66, 12.33)	-	9.87 (8.55, 11.12)	-
R	0.76 (0.64, 0.84)	-	0.80 (0.69, 0.87)	-
CCC	0.70 (0.58, 0.77)	-	0.74 (0.64, 0.80)	-
R^2^	0.56 (0.39, 0.66)	-	0.62 (0.43, 0.70)	-
Proportional bias: intercept, %	24.58 (13.91, 35.24)	<0.001	21.11 (11.29, 30.93)	<0.001
Proportional bias: slope	−0.44 (−0.61, −0.26)	<0.001	−0.41 (−0.58, −0.25)	<0.001
Magnitude-dependent variability: intercept, %	16.00 (9.99, 22.00)	<0.001	11.99 (5.50, 18.48)	<0.001
Magnitude-dependent variability: slope	−0.17 (−0.27, −0.07)	0.001	−0.11 (−0.21, 0.00)	0.057

Agreement metrics between AI-based and reference biplane LVEF estimates and OLS regression coefficients for proportional bias and magnitude-dependent variability for EchoNet and PanEcho. Agreement metric point values are computed across all videos curated, and 95% CIs computed using a patient-level percentile bootstrap, with patients resampled with replacement over 10,000 iterations. Regression coefficients and 95% CIs were estimated using cluster-robust standard errors with patients as clusters, and *P* values were based on 2-sided tests.

Abbreviations as in Table 1.

**Figure 4:**
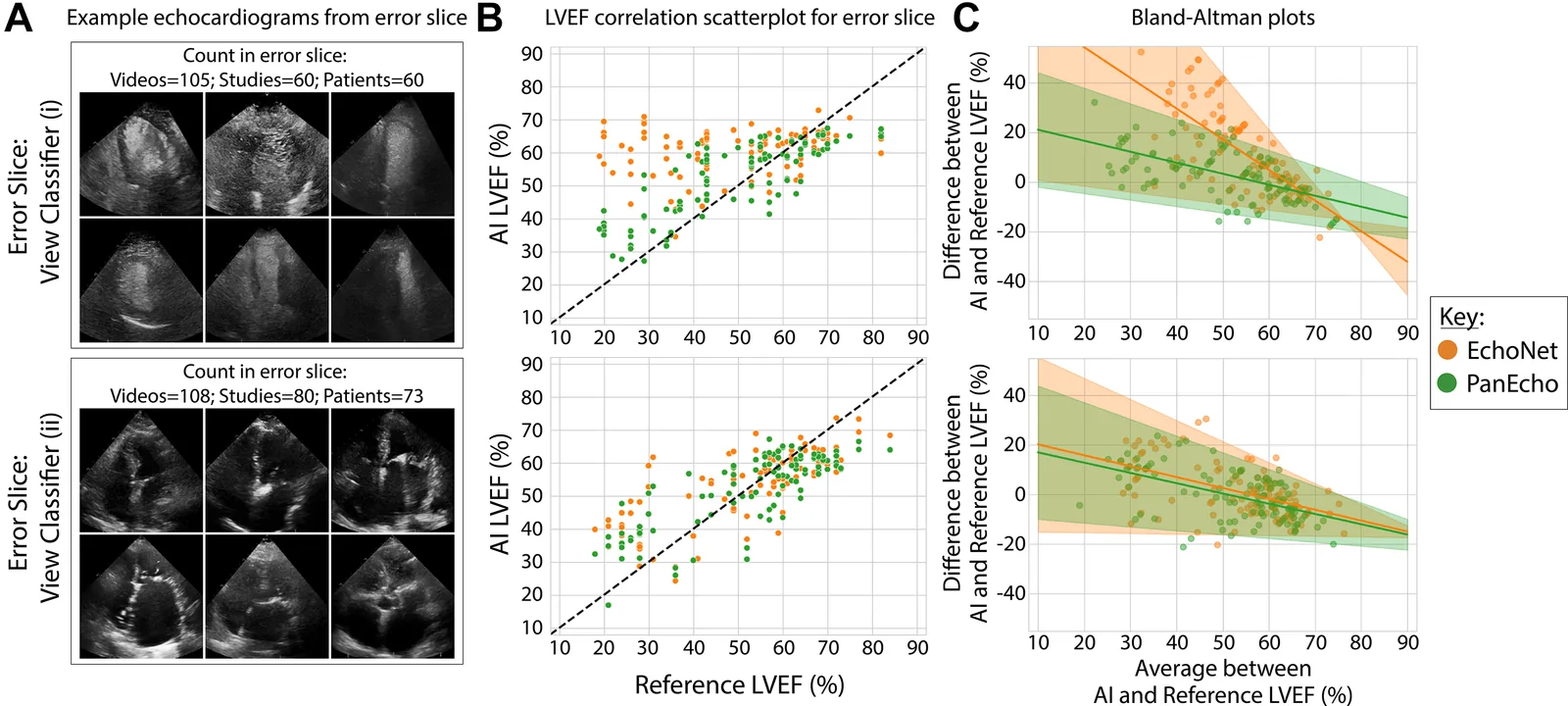
Left Ventricular Ejection Fraction Estimation Performance for Echocardiograms From View Classifier Error Slices Rows correspond to the 2 view-classifier error slices highlighted in Figure 3A (top, slice i; bottom, slice ii). (A) Example echocardiograms from each slice, with the number of videos, studies, and patients in-slice indicated above. (B) Scatterplots of artificial intelligence–based vs reference left ventricular ejection fraction estimates, with the dashed line representing perfect correlation (y = x). (C) Bland-Altman plots: the solid line is the proportional-bias regression (difference regressed on average) and the shaded band representing 95% limits of agreement from magnitude-dependent variability regression analysis. Orange and green colored points and bands denote EchoNet and PanEcho, respectively. Abbreviations as in Figure 1.

Table 4 summarizes the differences in patient- and study-level characteristics for studies inside vs outside the 2 shared view classifier error slices (Supplemental Tables 8 to 10 summarize analogous results for identified error slices using other feature spaces). Studies inside the contrast-enhanced slice had higher odds of coming patients with obesity (OR: 2.57 [1.53, 4.32], *P* < 0.001), lower odds of acquisition on Vivid S70 devices (OR: 0.58 [0.33, 1.00], *P* = 0.048), and lower reference biplane LVEF (mean difference [Δ]: −7.23 [−11.39, −3.07], *P* = 0.001). Studies inside the poor-quality, ambiguous, or mislabeled A4C slice had older patients (Δ: 7.67 [4.76, 10.58], *P* < 0.001), lower odds of male sex (OR: 0.57 [0.36, 0.92], *P* = 0.021), lower odds of obesity (OR: 0.51 [0.29, 0.91], *P* = 0.022), and lower reference LVEF (Δ: −3.63 [−7.04, −0.22], *P* = 0.037).

**Table 4:** Patient- and Study-Level Characteristics of Studies Inside Versus Outside View Classifier Error Slices

	In-Slice	Out-Slice	Effect (95% CI)	*P* Value
Error slice: contrast-enhanced views (Figure 3Ai)				
Age, y—mean (SD)	70.1 (12.8)	71.6 (13.2)	−1.50 (−4.85, 1.85)	0.381
Sex, male vs female—n (%)	28 (47.5)	603 (47.6)	0.99 (0.59, 1.68)	0.979
Race, White vs non-White—n (%)	26 (81.2)	476 (69.8)	1.88 (0.76, 4.63)	0.173
Obesity, BMI ≥30 kg/m^2^—n (%)	31 (51.7)	380 (29.4)	2.57 (1.53, 4.32)	<0.001
Ultrasound device: Vivid E95 vs others—n (%)	35 (58.3)	608 (47.0)	1.58 (0.93, 2.67)	0.089
Ultrasound device: Vivid S70 vs others—n (%)	20 (33.3)	601 (46.5)	0.58 (0.33, 1.00)	0.048
Ultrasound device: Vivid E90 vs others—n (%)	5 (8.3)	84 (6.5)	1.31 (0.51, 3.36)	0.576
Reference biplane LVEF, %—mean (SD)	51.5 (16.2)	58.7 (13.0)	−7.23 (−11.39, −3.07)	0.001
Error slice: poor quality, ambiguous, or mislabeled A4C views (Figure 3Aii)				
Age, y—mean (SD)	78.7 (12.8)	71.1 (13.1)	7.67 (4.76, 10.58)	<0.001
Sex, male vs female—n (%)	28 (35.0)	603 (48.4)	0.57 (0.36, 0.92)	0.021
Race, White vs non-White—n (%)	36 (66.7)	466 (70.6)	0.83 (0.46, 1.50)	0.543
Obesity, BMI ≥30 kg/m^2^—n (%)	15 (18.8)	396 (31.1)	0.51 (0.29, 0.91)	0.022
Ultrasound device: Vivid E95 vs others—n (%)	35 (43.8)	608 (47.8)	0.85 (0.54, 1.34)	0.487
Ultrasound device: Vivid S70 vs others—n (%)	41 (51.2)	580 (45.6)	1.26 (0.80, 1.97)	0.323
Ultrasound device: Vivid E90 vs others—n (%)	4 (5.0)	85 (6.7)	0.74 (0.26, 2.06)	0.559
Reference biplane LVEF, %—mean (SD)	55.0 (15.2)	58.6 (13.1)	−3.63 (−7.04, −0.22)	0.037

For each characteristic, the table reports its association with slice membership: mean differences (in-slice minus out-of-slice) for continuous variables (age, reference biplane LVEF), and OR for categorical variables (sex, obesity [BMI ≥30 kg/m^2^], ultrasound device), each with 95% CIs and 2-sided *P* values. Estimates derive from regressions with patient-clustered robust standard errors at the study level. A study was considered in-slice if at least one of its videos was in-slice. Studies with unknown characteristic data are not included in the regressions.

A4C = apical-4-chamber; BMI = body mass index; LVEF = left ventricular ejection fraction.

### Selective estimation results

Table 5 and Figure 5 present the selective estimation metrics and risk-coverage curves, respectively, for each combination of LVEF estimation model and deep feature representation (Supplemental Table 11 presents the differences between AURC and no-selection MAE). Selective estimation using EchoNet-Feats generally outperformed all other feature spaces: for both LVEF estimation models, it consistently achieved the lowest selective risk across varying coverage levels (EchoNet and PanEcho, R@C = 20%: 3.93 [3.07, 4.97] and 4.45 [3.36, 5.65]; R@C = 50%: 5.17 [4.47, 5.89] and 6.06 [5.21, 6.86]; R@C = 90%: 5.62 [5.05, 6.20] and 5.76 [5.14, 6.34]; AURC: 5.04 [4.45, 5.66] and 5.21 [4.54, 5.92]), and the highest study coverage at selective risk of 5 LVEF points (EchoNet and PanEcho, C@R = 5: 43.4 [26.2, 88.6] and 35.3 [2.1, 81.5]). Relative to the no-selection (naive aggregation) baseline (EchoNet and PanEcho, MAE: 6.08 [5.55, 6.64] and 5.75 [5.21, 6.30]), for EchoNet, selective estimation using only EchoNet-Feats and view classifier features significantly reduced risk averaged across coverage levels (EchoNet-Feats and view classifier, AURC minus no-selection MAE [Δ]: −1.04 [−1.54, −0.55] and −0.55 [−1.06, −0.02]), whereas for PanEcho, only EchoNet-Feats reduced coverage-averaged risk (Δ: −0.54 [−0.98, −0.07]).

**Table 5:** Selective Estimation Performance on the Held-Out Test Set

	AURC, LVEF Points	R@C = 20%, LVEF Points	R@C = 50%, LVEF Points	R@C = 90%, LVEF Points	C@R = 5 LVEF Points, %
EchoNet					
View classifier	5.53 (4.92, 6.19)	5.28 (4.17, 6.21)	5.34 (4.72, 6.11)	5.92 (5.35, 6.58)	24.8 (0.0, 66.1)
LV segmentator	6.33 (5.52, 7.23)	6.81 (5.29, 8.76)	5.99 (5.27, 6.88)	6.18 (5.59, 6.85)	0.0 (0.0, 40.7)
EchoNet-Feats	5.04 (4.45, 5.66)	3.93 (3.07, 4.97)	5.17 (4.47, 5.89)	5.62 (5.05, 6.20)	43.4 (26.2, 88.6)
PanEcho-Feats	5.90 (5.24, 6.58)	5.90 (4.76, 7.11)	5.80 (5.08, 6.55)	5.99 (5.42, 6.66)	8.4 (0.0, 65.9)
PanEcho					
View classifier	5.58 (4.85, 6.34)	5.47 (4.07, 6.64)	5.76 (4.77, 6.55)	5.82 (5.20, 6.48)	23.8 (0.0, 75.9)
LV segmentator	6.06 (5.32, 6.85)	6.41 (4.80, 7.78)	6.06 (5.21, 6.86)	6.04 (5.48, 6.66)	2.1 (0.0, 42.6)
EchoNet-Feats	5.21 (4.54, 5.92)	4.45 (3.36, 5.65)	5.08 (4.38, 5.90)	5.76 (5.14, 6.34)	35.3 (2.1, 81.5)
PanEcho-Feats	5.75 (5.11, 6.40)	5.58 (4.47, 6.82)	5.62 (4.85, 6.41)	6.02 (5.38, 6.62)	5.9 (0.7, 64.7)

Point values are computed across all videos in the held-out test set, and sets are 95% CIs computed using a patient-level percentile bootstrap, with patients found in the test-set resampled with replacement over 10,000 iterations.

AURC = area under risk coverage curve; C@R = coverage at risk; LV = left ventricle; R@C = risk at coverage.

**Figure 5:**
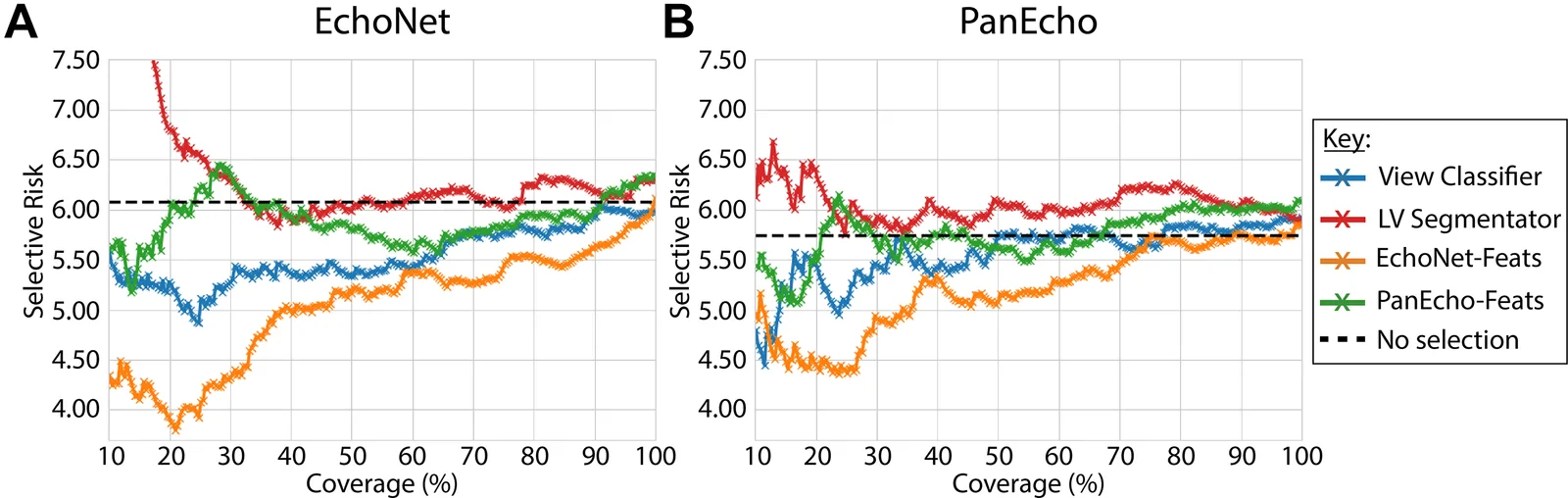
Risk-Coverage Curves for Selective Estimation of Left Ventricular Ejection Fraction Risk-coverage curves for selective estimation of left ventricular ejection fraction with (A) EchoNet and (B) PanEcho using the 4 deep feature representations on the held-out test set (blue: view classifier, red: left ventricle segmentator, orange: EchoNet-Feats, green: PanEcho-Feats). Each curve traces the selective risk—the mean absolute error between artificial intelligence–based and reference left ventricular ejection fraction over the retained studies—against coverage, the proportion of studies retained, as the abstention threshold (ie, maximum allowed misinterpretation risk) is varied. The black dashed line indicates the mean absolute error of the no-selection (naive aggregation) baseline. Lower curves indicate better selective estimation performance. Abbreviation as in Figure 3.

## Discussion

In this study, we investigated the utility of deep feature representations of echocardiograms to enable guardrails for echocardiography AI analysis algorithms. Specifically, we studied their effectiveness in error slicing, the task of identifying groups of echocardiograms sharing similar characteristics that result in large AI errors, and selective estimation, the task of excluding AI-generated estimates from high misinterpretation risk echocardiograms, in LVEF estimation from A4C videos using 2 validated DL-based models, EchoNet and PanEcho. We evaluated 4 deep feature representations derived from a view classifier, an LV segmentation model, and 2 LVEF estimation models. We conducted our experiments using retrospective data curated from the MIMIC-IV-ECHO database, which is external to the development data of both estimation models and heterogeneous with respect to image quality, acquisition protocol, and patient characteristics.

Consistent with findings from Pasdeloup et al,^17^ our overall evaluation reveals that both EchoNet and PanEcho exhibit significant errors and biased performance across key LVEF ranges. Specifically, both models are proportionally biased and have magnitude-dependent variability (ie, heteroskedasticity), systematically overestimating LVEF and exhibiting greater error variance in studies with reference LVEF <40% (Table 2, Figure 2). This has clear clinical implications: systematic overestimation in patients with reduced LVEF increases the risk of false negatives in heart failure with reduced ejection fraction diagnoses. Nevertheless, PanEcho consistently achieved greater agreement with the reference LVEF than EchoNet (EchoNet minus PanEcho [Δ], ΔMAE: 0.31 [0.07, 0.55]; ΔR^2^: −0.07 [−0.11, −0.03]; Δκ_w_: −0.06 [−0.09, −0.02]), suggesting that large-scale foundation models may offer inherent advantages in generalization for echocardiographic interpretation.

Our error slicing results show that both LVEF estimation models systematically underperform on poor-quality views of the LV (ie, poor endocardial border delineation or ambiguous A4C views) (Figure 3), a finding in line with prior studies.^1^^,^^11^^,^^25^ However, a novel finding of our study is the degraded performance of both models on contrast-enhanced echocardiograms, which is much more apparent for EchoNet than for PanEcho (Figure 4, top row). We believe this error slice ultimately stems from the training data used for model development: the videos used to train EchoNet do not contain contrast-enhanced echocardiograms, whereas those used to train PanEcho likely do but only represent a small proportion of the entire data set. We emphasize that the former conclusion is only possible because the EchoNet training data is publicly available. Without a nuanced knowledge of the training data used, practitioners applying AI models may fail to recognize which echocardiograms fall outside the model’s domain. Consequently, our proposed error slicing method enables echocardiography AI users to understand the strengths and limitations of models better when applied to their data, and guide them toward safer practices for AI usage.

Nevertheless, these error slice findings are particularly concerning given that poor-quality views and the use of contrast agents are often correlated with important patient subpopulations, including obese patients who are at higher risk of cardiovascular disease.^53^ Our patient- and study-level analyses corroborate that studies containing contrast-enhanced videos were more frequently acquired from obese patients (OR: 2.57 [1.53, 4.32], *P* < 0.001), whereas studies containing poor-quality, ambiguous, or mislabeled A4C views were more frequently acquired from older patients (mean age difference: 7.67 [4.76, 10.58], *P* < 0.001). Even more concerning, both error slices were associated with lower reference LVEF studies (contrast-enhanced and poor-quality A4C mean LVEF differences: −7.23 [−11.39, −3.07], *P* = 0.001, and −3.63 [−7.04, −0.22], *P* = 0.037), yet both models systematically overestimated LVEF (Figure 4). While we acknowledge that these findings do not establish causal associations and are specific to the studied MIMIC-IV-ECHO cohort, the clinical implications remain significant: error slices appear to be associated with higher-risk populations where accurate assessment is most critical.

The selective estimation results suggest that EchoNet-Feats offer a promising deep feature representation for effective guardrail construction. Across both models, EchoNet-Feats achieved the best selective estimation performance (EchoNet and PanEcho, AURC: 5.04 [4.45, 5.66] and 5.21 [4.54, 5.92]; C@R = 5: 43.4 [26.2, 88.6] and 35.3 [2.1, 81.5]). While prior studies have established that deep models can encode their own predictive uncertainty,^54^, ^55^, ^56^ it is noteworthy that features derived from an independent model can capture the failure modes of the other, suggesting that EchoNet possesses a generalizable latent representation of LVEF estimation difficulty for input echocardiograms. Nevertheless, selective estimation with PanEcho was generally less beneficial: using EchoNet-Feats, the measured improvement was smaller for PanEcho compared to EchoNet (EchoNet vs PanEcho, AURC minus no-selection MAE: −1.04 [−1.54, −0.55] vs −0.54 [−0.98, −0.07]). This suggests the inherent difficulty of estimating the misinterpretation risk of foundation models, whose errors may be more diffusely distributed across feature space rather than concentrated in identifiable clusters. Because foundation models are trained on diverse, large-scale data, their failure modes may be less systematic and therefore harder to anticipate with external feature representations alone. Notably, even PanEcho's own self-derived features (PanEcho-Feats) did not yield effective guardrails, indicating that the model's internal representations do not reliably separate accurate from inaccurate estimates. These findings highlight that selective estimation strategies effective for single-task models may not transfer directly to foundation models and that developing guardrail approaches tailored to the distributional properties of multitask architectures remains an important direction for future work.

Overall, our proposed error slicing and selective estimation demonstrated that deep feature representations can be used to identify failure modes of LVEF estimation models and automatically exclude misinterpretation-prone echocardiograms from AI analysis, offering 2 complementary guardrail mechanisms. First, identifying failure modes by error slicing gives practitioners an inspectable account of where an AI model underperforms on their own data, expressed as example videos. These findings can inform local governance policy on which studies are safe for AI interpretation, as well as guide internal efforts for model fine-tuning. Second, the proposed selective estimation method provides an automated quality control mechanism for AI oversight that leverages model- and data distribution-specific features learned from prior LVEF errors. Practitioners can set the abstention threshold according to their tolerance for measurement error and available reader capacity, with the risk-coverage curve quantifying the resulting trade-off (Figure 5). By reframing quality control as a process tailored to the deployed model and local data distribution rather than as a separate prefiltering task using hand-crafted image quality metrics, as is common with previous approaches,^25^^,^^26^ our framework provides a data-driven approach to building hospital-specific oversight mechanisms without requiring additional image quality annotations.

### Study limitations

Our analysis is limited to a retrospective evaluation on the MIMIC-IV-ECHO data set, which, although large and diverse, represents a single institution and may not fully reflect data distributions at other institutions. The cohort is derived from patients who appear in the MIMIC-IV clinical database,^57^ which contains data from patients admitted to the emergency department or intensive care unit between 2008 and 2019. This feature of the observed cohort should be considered when interpreting our specific findings, particularly regarding patient- and study-level characteristics within error slices. Furthermore, prospective multisite validation is necessary to support the generalization of the proposed error slicing and selective estimation methodology. Additionally, the reference biplane LVEF was extracted from measurements obtained during routine clinical care rather than standardized core laboratory measurements and is therefore subject to interobserver variability, which may impact observed agreement metrics, error distributions, and identified error slices. This variability is particularly important for studies with arrhythmias, for which no inclusion or exclusion criteria were applied in this study. Furthermore, while our proposed methodology for error slicing and selective estimation is general and can be expanded to alternative echocardiographic measurements and AI models, our analysis is limited to LVEF estimation from 2 DL-based models. Moreover, although PanEcho can estimate LVEF for alternative views, we limited our analysis to A4C views.

Finally, our proposed methodology assumes that echocardiography AI analysis algorithms systematically fail, that is, semantically or perceptually similar echocardiograms exhibit similar error profiles. However, this is not guaranteed by DL-based AI models, as prior literature has shown that neural networks are susceptible to large changes in their outputs with small, imperceptible modifications to their inputs, known as adversarial attacks.^58^, ^59^, ^60^ In cases where model failures are sporadic rather than systematic, feature-based guardrails may fail to identify individual high-risk AI estimates, underscoring the need for complementary uncertainty estimation approaches.

## Conclusions

We presented oversight strategies for echocardiography AI that leverage deep feature representations to identify model-specific errors and automatically flag records likely to yield unreliable AI-generated estimates. By evaluating 2 established DL-based LVEF estimation models on a heterogeneous external database, we uncovered key insights into the failure modes of the models and demonstrated that our proposed selective estimation method can improve LVEF estimation performance by abstaining from high-risk records. Our work takes an important step toward the safe and reliable deployment of AI systems used in automated echocardiography analysis and interpretation, helping bridge the gap between AI research and clinical translation.

Perspectives **COMPETENCY IN MEDICAL KNOWLEDGE:** This study suggests that AI models for echocardiographic LVEF estimation tend toward outputting values that appear normal (50% to 70%) in atypical or noisy images, such as those with poor acoustic windows or those acquired with contrast agents, independent of the patient’s true LVEF. Consequently, this systematic bias may lead to a higher false negative rate for identifying patients with reduced LVEF.

**COMPETENCY IN SYSTEMS-BASED PRACTICE:** As echocardiography AI tools are increasingly integrated into clinical workflows, institutions must implement quality assurance and oversight mechanisms that account for site-specific data characteristics, including ultrasound vendor differences, sonographer expertise, and patient demographics. The proposed approach presented in this study can adapt to local conditions without manual specification of echocardiography AI failure modes or image quality, instead relying on retrospective performance validation.

**COMPETENCY IN PATIENT CARE:** The proposed selective estimation approach can enable clinicians to selectively apply manual oversight for echocardiographic interpretation where it is most needed, rather than relying on blanket acceptance or rejection of automated results.

**TRANSLATIONAL OUTLOOK:** Prospective, multisite validation is needed to confirm that the error slicing and selective estimation methods generalize beyond the single-center MIMIC-IV-ECHO data set. Furthermore, because the framework assumes systematic rather than sporadic failure patterns, complementary uncertainty estimation methods should be explored to address adversarial or idiosyncratic errors. Future research should evaluate the integration of these guardrail mechanisms into real-time clinical workflows, assess their impact on diagnostic accuracy and clinician decision-making in prospective studies, and extend the methodology to additional echocardiographic measurements and AI architectures.

## Funding support and author disclosures

The authors have reported that they have no relationships relevant to the contents of this paper to disclose.
