# Supplementary material for: Algorithmic Guardrails for Echocardiography AI: Deep Feature Representations for Error Slicing and Selective LVEF Estimation
Source: JACC Adv. 2026 Oct 13;5(11):103333. doi: 10.1016/j.jacadv.2026.103333 (PMC13685970; doi:10.1016/j.jacadv.2026.103333)
Supplement: Supplementary file 1 [file mmc1.pdf]

Supplementary Materials

Manuscript Title: Algorithmic Guardrails for Echocardiography AI: Deep Feature Representations for Error Slicing and Selective LVEF Estimation

Krish Kabra, MS[1], Sadeer Al-Kindi, MD[2], Guha Balakrishnan PhD[1]

[1]Department of Electrical and Computer Engineering, Rice University, Houston, TX 77005
[2]Center for Cardiovascular Computational and Precision Health, Department of Cardiology, DeBakey Heart and Vascular Center, Houston Methodist, Houston, TX 77030

Table of Contents

Supplementary Methods 3

MIMIC-IV-ECHO Data Curation Overview 3

Echocardiography Video Preprocessing 3

LVEF Estimation Model Inference 3

Consensus Clustering Pipeline for Error Slicing 3

Clustering and Error Slicing Stability Analysis 4

Supplementary References 5

Supplementary Figures 6

Supplementary Figure 1. Data curation pipeline 6

Supplementary Figure 2. Additional error slices identified by deep feature representations of echocardiograms 7

Supplementary Figure 3. LVEF estimation performance for echocardiograms from LV segmentator error slices. 8

Supplementary Figure 4. LVEF estimation performance for echocardiograms from EchoNet-Feats error slices. 9

Supplementary Figure 5. LVEF estimation performance for echocardiograms from PanEcho-Feats error slices. 10

Supplementary Tables 11

Supplementary Table 1. MIMIC-IV-ECHO dataset characteristics 11

Supplementary Table 2. Stability of consensus clustering and error slicing 12

Supplementary Table 3. Hyperparameter grid-search for selective estimation 13

Supplementary Table 4. Differences in mean absolute errors between in-slice and out-of-slice echocardiograms for all identified error slices 14

Supplementary Table 5. LVEF estimation evaluation metrics on echocardiograms from LV segmentator error slices 15

Supplementary Table 6. LVEF estimation evaluation metrics on echocardiograms from EchoNet-Feats error slices 16

Supplementary Table 7. LVEF estimation evaluation metrics on echocardiograms from PanEcho-Feats error slices 17

Supplementary Table 8. Patient- and study-level characteristics of studies inside versus outside LV segmentator error slices 18

Supplementary Table 9. Patient- and study-level characteristics of studies inside versus outside EchoNet-Feats error slices 20

Supplementary Table 10. Patient- and study-level characteristics of studies inside versus outside PanEcho-Feats error slices 22

Supplementary Table 11. Differences between selective estimation AURC and no-selection MAE 24

Supplementary Table 12. TRIPOD+AI Checklist 25

Supplementary Methods

MIMIC-IV-ECHO Data Curation Overview

The MIMIC-IV-ECHO database[1] contains 525,422 echocardiogram records (289,879 video records and 235,543 image records) from 7,243 transthoracic echocardiography (TTE) studies collected at the Beth Israel Deaconess Medical Center (BIDMC) of 4,579 distinct patients admitted between 2017-2019. A portion of these studies is linked to a database of structured measurements containing cardiologist-recorded echocardiographic measurements, including chamber sizes and volumes, systolic and diastolic function, valvular morphology and function, and Doppler-derived hemodynamics, derived from a much larger cohort observed in MIMIC-IV clinical database[2,3]. For the purposes of this study, we extracted and linked left ventricular ejection fraction (LVEF) measurements obtained by Simpson's biplane method (identified with key "biplane_lvef") to 1,521 studies of 1,208 patients. From these studies, after preprocessing the echocardiogram videos (see section below), we curated a final dataset consisting of 4,010 apical-4-chamber (A4C) videos from 1,353 studies of 1,208 patients. An overview of the data curation pipeline is provided in Supplementary Figure 1.

Echocardiography Video Preprocessing

Our investigations focused on echocardiography artificial intelligence (AI) analysis algorithms used for LVEF estimation from A4C viewpoints. To select A4C video records from the database, we use a publicly available pretrained view classifier[4] that can distinguish between 23 fine-grained TTE views. We computed the average view probability across all frames of each video and selected videos with a maximum view probability corresponding to either the "A4C" or "A4C LAOCC" views. We then processed the A4C videos for input into the AI analysis algorithms by masking pixels outside the central image area, taking a central square crop of the video, and converting the videos to grayscale. This processing is identical to established procedures of prior works[5,6], ensuring that input formatting was consistent with the AI analysis algorithms' original training pipelines. Finally, to ensure each video contained sufficient cardiac cycles for assessment, we rejected videos with fewer than 15 frames (~0.5 seconds). This process yielded 18,214 A4C videos from 6,259 studies of 4,160 patients.

LVEF Estimation Model Inference

EchoNet[7] and PanEcho[8] estimate LVEF from standardized video clips that typically capture one cardiac cycle. However, their input specifications differ: EchoNet processes 32-frame clips, whereas PanEcho uses 16-frame clips. Because echocardiogram videos have variable durations and often contain multiple cardiac cycles, we subdivided each A4C video into overlapping clips using a single-frame stride to enable consistent inference across both AI analysis algorithms. For videos with fewer than the required number of frames (i.e., <16 or <32 frames), blank frames were appended to satisfy input requirements. Final LVEF estimates were obtained by averaging predictions across all sampled clips.

Consensus Clustering Pipeline for Error Slicing

To perform error slicing using a given feature representation, we first identified a stable clustering of the embedded echocardiograms using a consensus clustering procedure based on the evidence-accumulation framework[9]. The procedure consisted of three steps: (i) *generation of base clusterings*, where we generated an ensemble of clusterings using a combination of the Uniform Manifold Approximation[10] (UMAP) dimensionality reduction algorithm and the Hierarchical Density-Based Spatial Clustering of Applications with Noise[11] (HDBSCAN) clustering algorithm across a sweep of hyperparameters and random seeds, (ii) *co-association matrix construction*, where we aggregated the base clusterings into a matrix encoding the co-clustering frequency of each pair of echocardiograms, and (iii) *consensus clustering*, where we derived a single set of consensus cluster assignments by clustering the co-association matrix. All experiments used umap-learn v0.5.12 and hdbscan v0.8.43.

Generation of base clusterings. Given the high dimensionality of deep feature embeddings, we follow prior work[12] that uses UMAP to reduce dimensionality before clustering with HDBSCAN. To avoid relying on any one choice of UMAP hyperparameters, we swept the two settings with the largest effect on embedding structure: embedding dimensionality ($n_{components}$) and neighborhood size ($n_{neighbors}$), over the grid $n_{components} \in \{4, 8, 16\}$ and $n_{neighbors} \in \{10, 20, 50, 100\}$, yielding 12 hyperparameter configurations. The Euclidean distance metric was used throughout, and all remaining UMAP parameters were set to their default values. For each configuration, UMAP was run with 10 independent random seeds, producing a total of $K = 12 \times 10 = 120$ base clusterings per ensemble. HBSCAN clustering was performed using a minimum cluster size of 20, the Euclidean distance metric, and the leaf cluster selection method (which favors small homogeneous clusters over fewer large clusters that are

produced by the default excess of mass method). We denote the result of the kth base clustering by $\pi^k \in \mathbb{Z}^n$, which is an integer-valued vector of length n that assigns each echocardiogram a cluster label, with $\pi_i^k = -1$ reserved for echocardiograms assigned to the HDBSCAN noise label.

Co-association matrix construction. From the set of base clusterings $\{\pi^k\}_{k=1}^K$, we constructed a symmetric co-association matrix, $M \in [0,1]^{n \times n}$, whose entries encode the co-clustering frequencies per echocardiogram. Specifically, we define the pairwise co-clustering indicator:

$$\delta(\pi_i^k, \pi_j^k) = \begin{cases} 1, & \pi_i^k = \pi_j^k \text{ and } \pi_i^k \neq -1 \text{ and } \pi_j^k \neq -1 \\ 0, & \text{otherwise} \end{cases}$$

which equals 1 if and only if records i and j were assigned to the same non-noise cluster under base clustering π^k. Then, the co-association matrix is defined as:

$$M_{ij} = \frac{1}{K} \sum_{k=1}^{K} \delta(\pi_i^k, \pi_j^k)$$

Consensus clustering. Consensus clustering assignments were obtained by treating $(1 - M)$ as a precomputed pairwise distance (with diagonal set to zero) and applying HDBSCAN a second time with minimum cluster size of 20 and cluster selection epsilon as 0.1 (which clusters neighbors whose constituent echocardiograms co-clustered in at least 90% of base runs, preventing over-fragmentation of highly stable structure). Echocardiograms that remained unassigned after this step retained the noise label and were excluded from the downstream error slice determination.

Clustering and Error Slicing Stability Analysis

To assess the stability of the clustering and subsequent error slicing assignments, the entire consensus clustering and error slicing procedures (see Methods, Error Slicing) were repeated 10 times for each feature representation, with all UMAP random seeds resampled at each iteration. We quantified stability using the mean adjusted Rand index[13] (ARI) across consensus cluster and error slicing labels produced by each pair of procedure repetitions. The ARI computes a similarity measure between two clusterings that is adjusted for random chance, with values ranging from 0 to 1: 0 indicates agreement no better than chance, and 1 indicates identical partitions. Supplementary Table 2 reports the mean and standard deviation of ARI scores across all 45 (10 choose 2) repetition pairs.

Supplementary References

1. Gow B, Pollard T, Greenbaum N, et al. MIMIC-IV-ECHO: Echocardiogram Matched Subset. doi:10.13026/EF48-V217

2. Johnson A, Bulgarelli L, Pollard T, Horng S, Celi LA, Mark R. MIMIC-IV. doi:10.13026/6MM1-EK67

3. Johnson AEW, Bulgarelli L, Shen L, et al. MIMIC-IV, a freely accessible electronic health record dataset. *Sci Data*. 2023;10(1):1. doi:10.1038/s41597-022-01899-x

4. Zhang J, Gajjala S, Agrawal P, et al. Fully Automated Echocardiogram Interpretation in Clinical Practice. *Circulation*. 2018;138(16):1623-1635. doi:10.1161/CIRCULATIONAHA.118.034338

5. Holste G, Oikonomou EK, Tokodi M, Kovács A, Wang Z, Khera R. Complete AI-Enabled Echocardiography Interpretation With Multitask Deep Learning. *JAMA*. 2025;334(4):306-318. doi:10.1001/jama.2025.8731

6. Holste G, Oikonomou EK, Mortazavi BJ, et al. Severe aortic stenosis detection by deep learning applied to echocardiography. *Eur Heart J*. 2023;44(43):4592-4604. doi:10.1093/eurheartj/ehad456

7. Ouyang D, He B, Ghorbani A, et al. Video-based AI for beat-to-beat assessment of cardiac function. *Nature*. 2020;580(7802):252-256. doi:10.1038/s41586-020-2145-8

8. Holste G, Oikonomou EK, Tokodi M, Kovács A, Wang Z, Khera R. Complete AI-Enabled Echocardiography Interpretation With Multitask Deep Learning. *JAMA*. 2025;334(4):306-318. doi:10.1001/jama.2025.8731

9. Fred ALN, Jain AK. Combining multiple clusterings using evidence accumulation. *IEEE Trans Pattern Anal Mach Intell*. 2005;27(6):835-850. doi:10.1109/TPAMI.2005.113

10. McInnes L, Healy J, Saul N, Großberger L. UMAP: Uniform Manifold Approximation and Projection. *J Open Source Softw*. 2018;3(29):861. doi:10.21105/joss.00861

11. Campello RJGB, Moulavi D, Sander J. Density-Based Clustering Based on Hierarchical Density Estimates. In: Pei J, Tseng VS, Cao L, Motoda H, Xu G, eds. *Advances in Knowledge Discovery and Data Mining*. Springer; 2013:160-172. doi:10.1007/978-3-642-37456-2_14

12. Sohoni N, Dunnmon JA, Angus G, Gu A, Ré C. No subclass left behind: fine-grained robustness in coarse-grained classification problems. In: *Proceedings of the 34th International Conference on Neural Information Processing Systems*. NIPS '20. Curran Associates Inc.; 2020:19339-19352. Accessed June 1, 2026. https://dl.acm.org/doi/10.5555/3495724.3497346

13. Hubert L, Arabie P. Comparing partitions. *J Classif*. 1985;2(1):193-218. doi:10.1007/BF01908075

Supplementary Figures

Supplementary Figure 1. Data curation pipeline

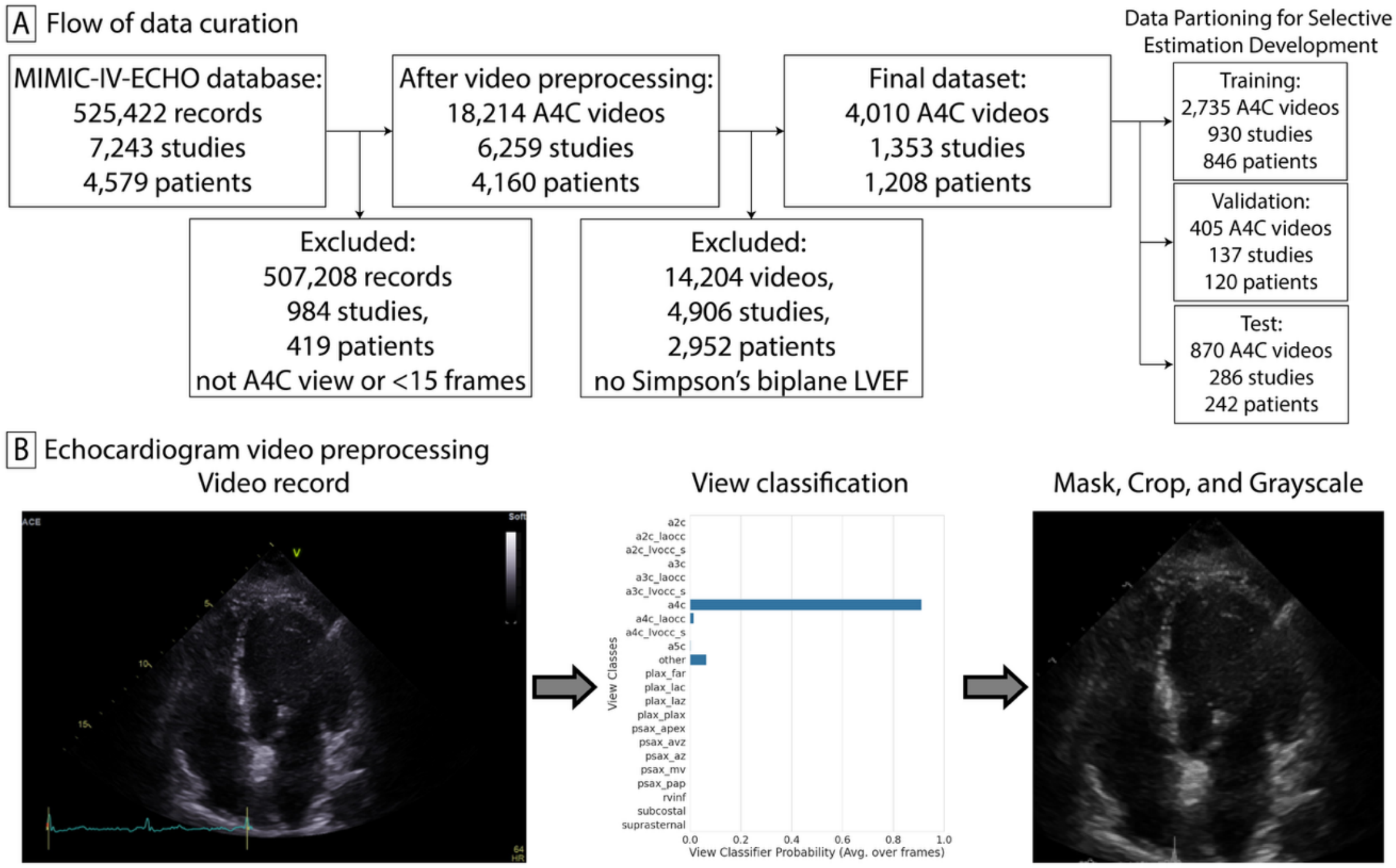

(A) Flow chart of the data curation process at each stage. (B) Overview of echocardiogram video preprocessing.

Supplementary Figure 2. Additional error slices identified by deep feature representations of echocardiograms

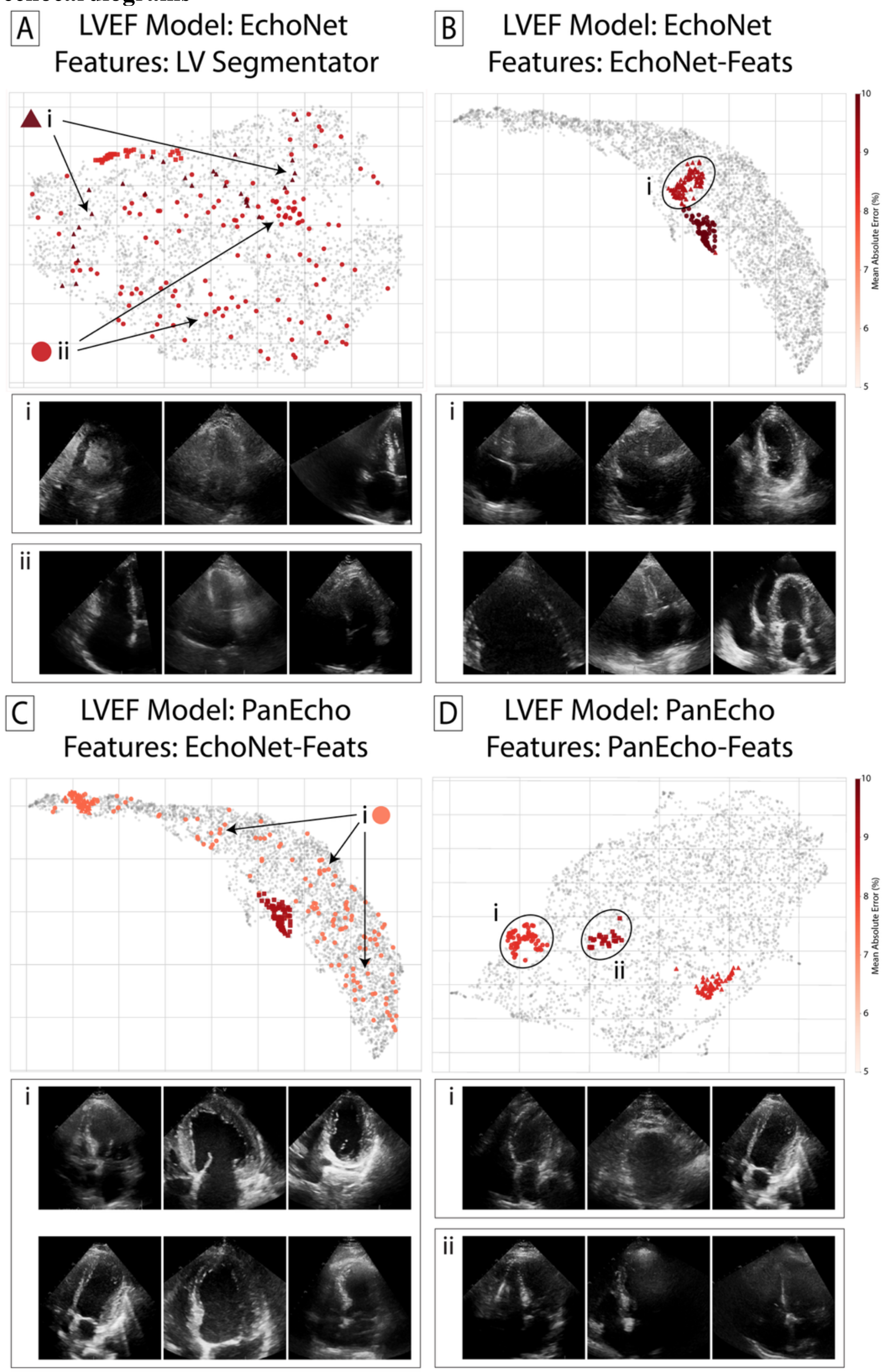

Supplementary Figure 3. LVEF estimation performance for echocardiograms from LV segmentator error slices.

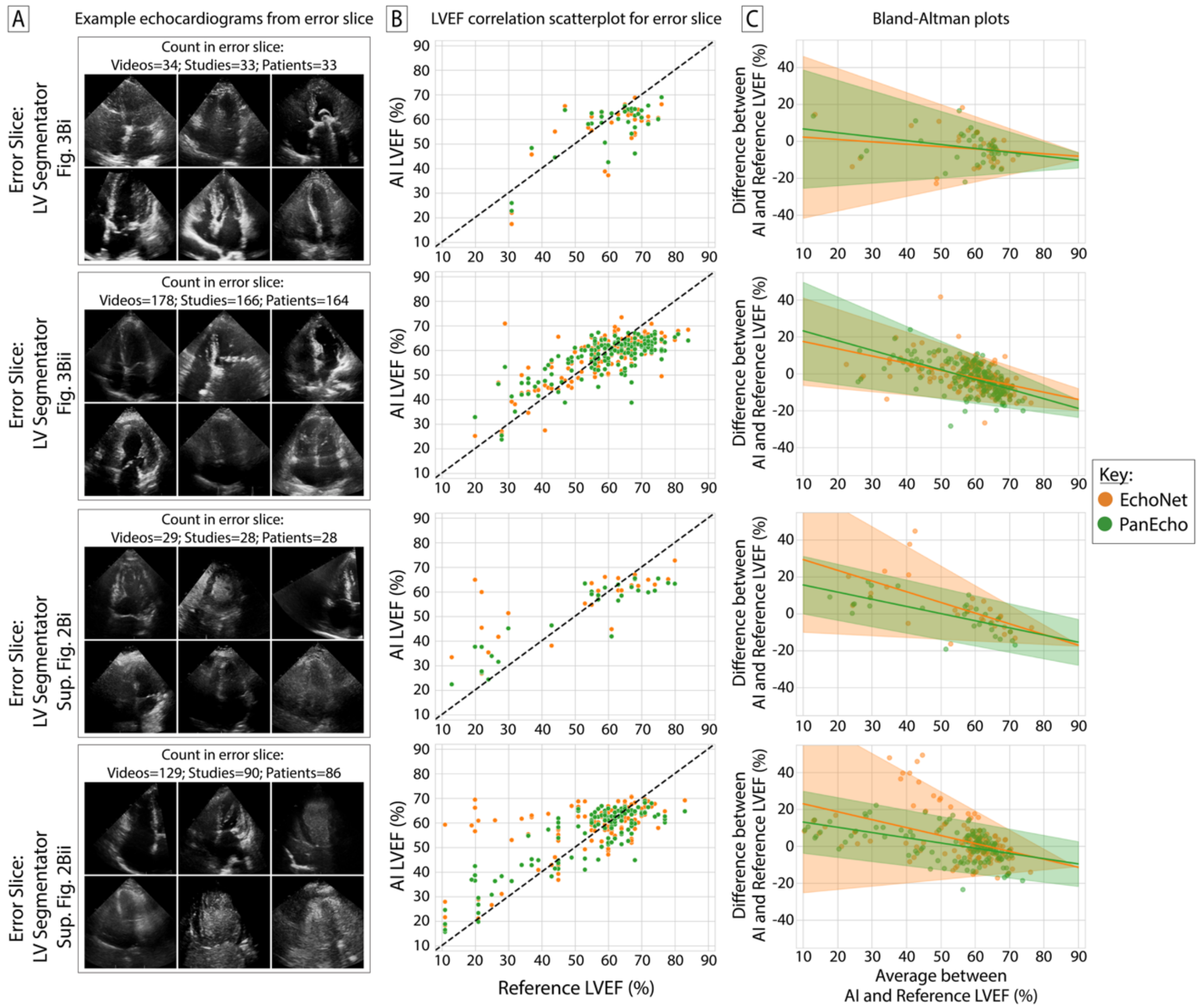

Supplementary Figure 4. LVEF estimation performance for echocardiograms from EchoNet-Feats error slices.

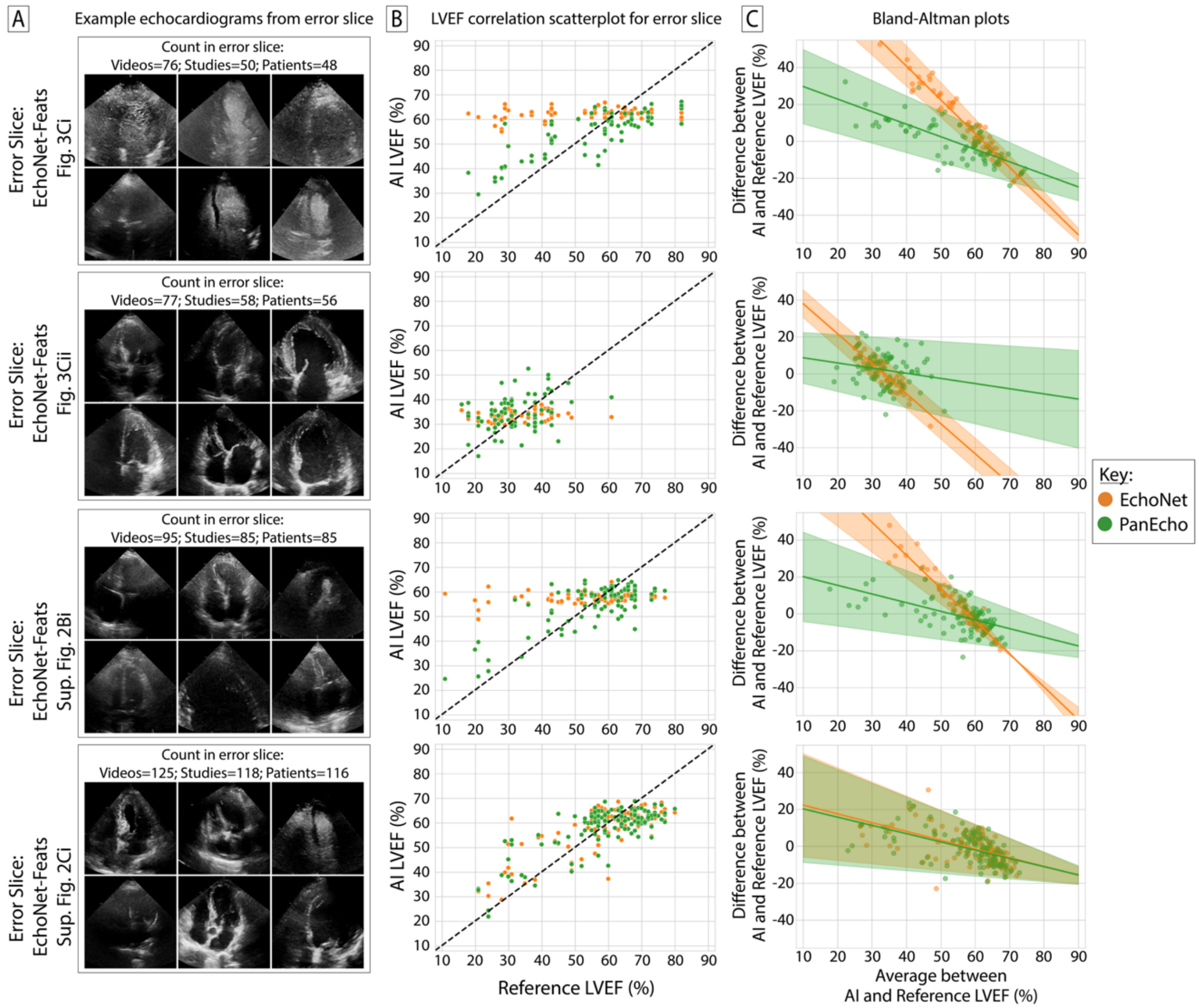

Supplementary Figure 5. LVEF estimation performance for echocardiograms from PanEcho-Feats error slices.

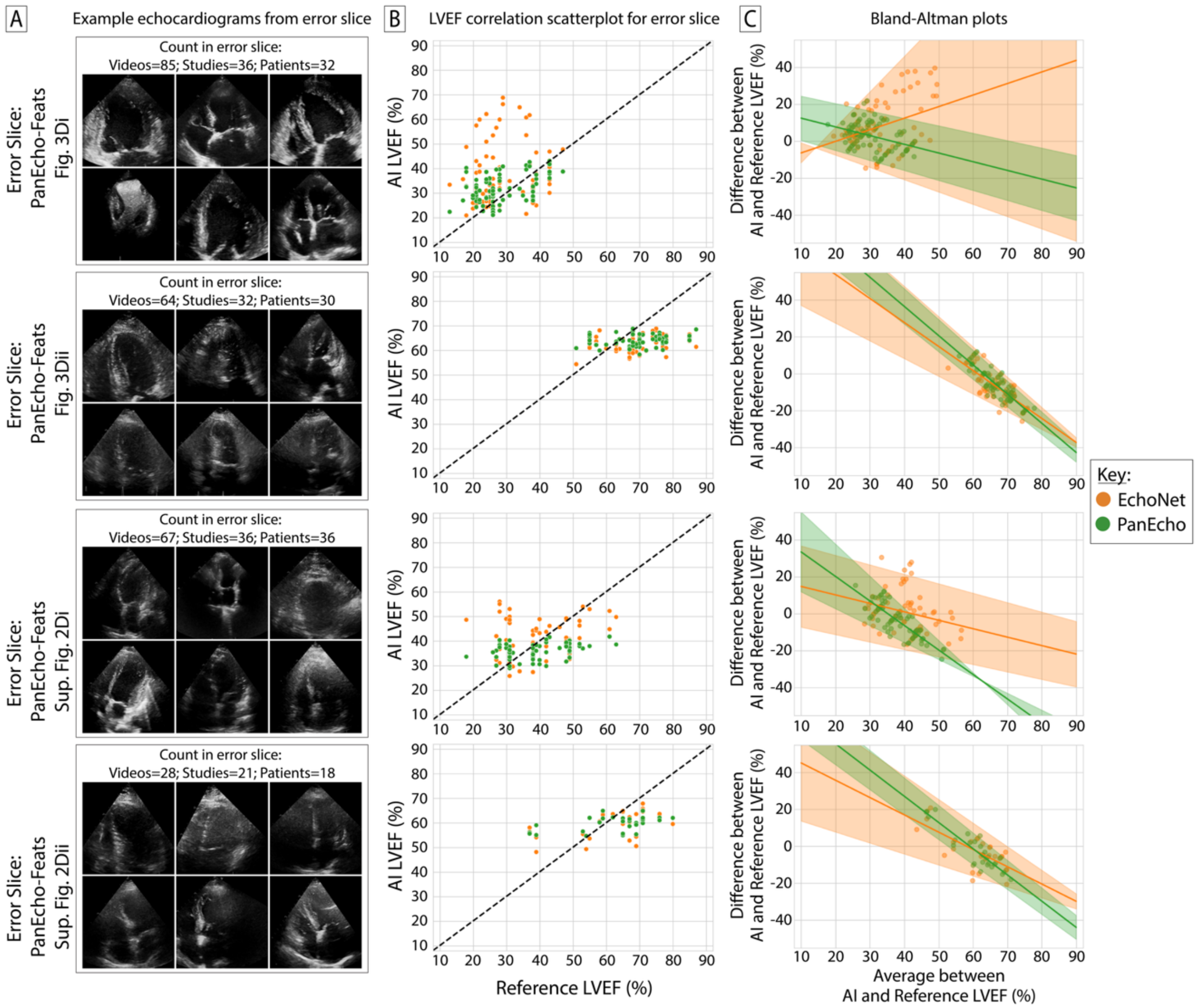

Supplementary Tables

Supplementary Table 1. MIMIC-IV-ECHO dataset characteristics

**Unique patients, No.**		1,208
**Unique studies, No.**		1,353
**Unique videos, No.**		4,010
**Patient Characteristics**		
Age, Mean (SD)		58 (13)
Sex, No. (%)	Male	563 (46.6)
	Female	617 (51.1)
	Other/Unknown	28 (2.3)
Race/Ethnicity, No. (%)	White	453 (37.5)
	Black	117 (9.7)
	Asian	24 (2.0)
	Hispanic/Latino	25 (2.1)
	Native American/Pacific Islander	2 (0.2)
	Other/Unknown	557 (46.1)
BMI, Mean (SD)		29.2 (15.3)
Obesity (BMI ≥30 kg/m^2), No. (%)		411 (30.4)
**Study Characteristics**		
Ultrasound Device Model, No. (%)	Vivid E95	643 (47.5)
	Vivid S70	621 (45.9)
	Vivid E90	89 (6.6)
LVEF, Mean (SD), %		58.4 (13.2)
LVEF categories, No. (%)	<40%	138 (10.2)
	40-50%	114 (8.4)
	>50%	1101 (81.4)

Patient age and BMI were unique to each study. Abbreviations: BMI = Body mass index; No. = Number of; SD = Standard deviation; LVEF = Left ventricular ejection fraction

Supplementary Table 2. Stability of consensus clustering and error slicing

Feature Space	Consensus Clustering ARI, Mean (SD)	Error Slicing ARI, Mean (SD	
		EchoNet	*PanEcho*
View classifier	0.60 (0.14)	0.95 (0.02)	0.90 (0.04)
LV Segmentator	0.59 (0.10)	0.60 (0.26)	0.80 (0.40)
EchoNet-Feats	0.68 (0.05)	0.48 (0.30)	0.60 (0.12)
PanEcho-Feats	0.63 (0.02)	0.70 (0.13)	0.75 (0.14)

Consensus clustering and error slicing stability were assessed by running the respective pipelines 10 times per feature representation and LVEF estimation model (note that consensus clustering depends only on the feature representation and does not vary across LVEF models). The table reports the mean and SD of the ARI computed across all 45 (10 choose 2) repetition pairs of clustering and error slicing labels. Abbreviations: ARI = adjusted Rand index; LV = left ventricle; SD = Standard deviation

Supplementary Table 3. Hyperparameter grid-search for selective estimation

Name of hyperparameter	Values
**UMAP**	
Number of components (n_components)	[2, 4, 8, 16, 32, 64, 128, 256]
Size of local neighborhood (n_neighbors)	[10, 20, 50, 100, 200]
Distance Metric (metric)	["euclidean", "manhattan", "cosine"]
**k-Nearest Neighbor Regression**	
Number of neighbors (n_neighbors)	[5:5:100]
Distance Metric (metric)	["euclidean", "manhattan", "cosine"]
Weighting (weights)	["uniform", "distance"]

Supplementary Table 4. Differences in mean absolute errors between in-slice and out-of-slice echocardiograms for all identified error slices

Error Slice		EchoNet		PanEcho	
**Feature Space**	**Reference**	**ΔMAE, % (95% CI)**	**P-Value**	**ΔMAE, % (95% CI)**	**P-Value**
View Classifier	Figure 3Ai	8.44 (3.97, 12.91)	<0.001	2.07 (0.48, 3.66)	0.005
View Classifier	Figure 3Aii	1.77 (0.27, 3.27)	0.010	2.13 (0.88, 3.38)	<0.001
LV Segmentator	Figure 3Bi	1.85 (-0.16, 3.87)	0.036	1.01 (-0.86, 2.87)	0.145
LV Segmentator	Figure 3Bii	-0.15 (-0.89, 0.60)	0.648	0.84 (0.07, 1.62)	0.017
LV Segmentator	Supplementary Figure 2Ai	3.80 (0.23, 7.36)	0.018	1.50 (-0.52, 3.52)	0.073
LV Segmentator	Supplementary Figure 2Aii	2.27 (-0.03, 4.57)	0.027	0.41 (-0.78, 1.61) (p=0.248)	0.248
EchoNet-Feats	Figure 3Ci	7.44 (3.84, 11.05)	<0.001	3.05 (1.23, 4.87)	0.001
EchoNet-Feats	Figure 3Cii	-0.06 (-1.28, 1.15)	0.541	1.26 (-0.01, 2.53)	0.026
EchoNet-Feats	Supplementary Figure 2Bi	2.63 (0.80, 4.45)	0.002	0.50 (-0.67, 1.67)	0.200
EchoNet-Feats	Figure 2Ci	0.45 (-0.50, 1.41)	0.176	1.09 (0.18, 1.99)	0.009
PanEcho-Feats	Figure 3Di	4.84 (1.60, 8.09)	0.002	0.29 (-1.32, 1.90)	0.363
PanEcho-Feats	Figure 3Dii	2.24 (0.14, 4.34)	0.018	2.17 (-0.07, 4.41)	0.029
PanEcho-Feats	Supplementary Figure 2Di	1.90 (-0.38, 4.17)	0.051	2.05 (0.29, 3.81)	0.011
PanEcho-Feats	Supplementary Figure 2Dii	1.53 (-1.17, 4.22)	0.133	2.95 (0.46, 5.44)	0.010

Estimated differences in mean absolute errors between in-slice and out-of-slice videos for all identified error slices. Error slices in the first column are referenced using those identified in Figure 3 and Supplementary Figure 2. Differences are estimated at the video level via OLS regression of each echocardiogram's absolute error on a binary indicator of error slice membership. P-values correspond to a one-sided test for a positive difference (ΔMAE > 0).

Supplementary Table 5. LVEF estimation evaluation metrics on echocardiograms from LV segmentator error slices

	EchoNet		PanEcho	
	**Value (95% CI)**	**P-value**	**Value (95% CI)**	**P-value**
Error Slice: Figure 3Bi				
MAE, %	8.46 (6.49, 10.48)	-	7.31 (5.56, 9.22)	-
RMSE, %	10.34 (8.26, 12.28)	-	9.07 (6.99, 11.03)	-
R	0.76 (0.37, 0.89)	-	0.82 (0.46, 0.92)	-
CCC	0.73 (0.31, 0.85)	-	0.78 (0.39, 0.88)	-
R^2	0.50 (-0.49, 0.74)	-	0.61 (-0.17, 0.81)	-
Proportional bias: Intercept, %	3.54 (-15.80, 22.88)	0.720	8.79 (-5.17, 22.75)	0.217
Proportional bias: Slope	-0.13 (-0.44, 0.18)	0.411	-0.21 (-0.43, 0.01)	0.061
Magnitude-dependent variability: Intercept, %	19.94 (13.41, 26.47)	<0.001	14.44 (7.72, 21.16)	<0.001
Magnitude-dependent variability: Slope	-0.21 (-0.32, -0.11)	<0.001	-0.14 (-0.25, -0.03)	0.010
Error Slice: Figure 3Bii				
MAE, %	6.48 (5.75, 7.26)	-	7.12 (6.35, 7.91)	-
RMSE, %	8.25 (7.03, 9.63)	-	8.76 (7.85, 9.66)	-
R	0.74 (0.60, 0.83)	-	0.72 (0.60, 0.80)	-
CCC	0.69 (0.55, 0.79)	-	0.63 (0.49, 0.73)	-
R^2	0.53 (0.33, 0.66)	-	0.47 (0.27, 0.59)	-
Proportional bias: Intercept, %	21.41 (11.96, 30.85)	<0.001	28.48 (16.86, 40.10)	<0.001
Proportional bias: Slope	-0.39 (-0.54, -0.24)	<0.001	-0.52 (-0.71, -0.34)	<0.001
Magnitude-dependent variability: Intercept, %	10.53 (4.98, 16.08)	<0.001	11.86 (5.02, 18.70)	0.001
Magnitude-dependent variability: Slope	-0.09 (-0.18, -0.00)	0.045	-0.11 (-0.22, 0.00)	0.050
Error Slice: Supplementary Figure 2Ai				
MAE, %	10.39 (7.00, 14.21)	-	7.80 (5.85, 9.93)	-
RMSE, %	14.74 (9.19, 19.65)	-	9.59 (7.50, 11.57)	-
R	0.75 (0.52, 0.92)	-	0.92 (0.83, 0.96)	-
CCC	0.64 (0.40, 0.81)	-	0.85 (0.74, 0.91)	-
R^2	0.49 (0.13, 0.74)	-	0.79 (0.62, 0.86)	-
Proportional bias: Intercept, %	35.14 (23.30, 46.98)	<0.001	19.49 (11.59, 27.38)	<0.001
Proportional bias: Slope	-0.58 (-0.76, -0.40)	<0.001	-0.39 (-0.53, -0.25)	<0.001
Magnitude-dependent variability: Intercept, %	17.94 (5.93, 29.95)	0.003	6.50 (2.72, 10.29)	0.001
Magnitude-dependent variability: Slope	-0.20 (-0.38, -0.02)	0.032	-0.02 (-0.08, 0.05)	0.631
Error Slice: Supplementary Figure 2Aii				
MAE, %	8.82 (6.61, 11.22)	-	6.72 (5.55, 7.93)	-
RMSE, %	13.29 (9.52, 16.85)	-	8.46 (7.12, 9.76)	-
R	0.69 (0.34, 0.83)	-	0.89 (0.73, 0.94)	-
CCC	0.63 (0.27, 0.78)	-	0.85 (0.69, 0.91)	-
R^2	0.43 (-0.00, 0.65)	-	0.77 (0.53, 0.86)	-
Proportional bias: Intercept, %	27.31 (14.03, 40.60)	<0.001	15.94 (9.78, 22.09)	<0.001
Proportional bias: Slope	-0.43 (-0.64, -0.22)	<0.001	-0.28 (-0.39, -0.18)	<0.001
Magnitude-dependent variability: Intercept, %	22.02 (11.74, 32.30)	<0.001	7.07 (3.03, 11.10)	0.001
Magnitude-dependent variability: Slope	-0.25 (-0.41, -0.08)	0.004	-0.02 (-0.09, 0.05)	0.503

Supplementary Table 6. LVEF estimation evaluation metrics on echocardiograms from EchoNet-Feats error slices

	EchoNet		PanEcho	
	**Value (95% CI)**	**P-value**	**Value (95% CI)**	**P-value**
Error Slice: Figure 3Ci				
MAE, %	13.93 (10.25, 17.58)	-	9.31 (7.55, 11.15)	-
RMSE, %	18.48 (14.45, 22.14)	-	11.08 (8.92, 13.12)	-
R	0.22 (-0.08, 0.48)	-	0.82 (0.72, 0.90)	-
CCC	0.05 (-0.02, 0.12)	-	0.68 (0.58, 0.76)	-
R^2	-0.15 (-0.59, 0.05)	-	0.59 (0.45, 0.67))	-
Proportional bias: Intercept, %	113.94 (104.47, 123.41)	<0.001	36.45 (28.23, 44.67)	<0.001
Proportional bias: Slope	-1.83 (-1.98, -1.68)	<0.001	-0.68 (-0.82, -0.54)	<0.001
Magnitude-dependent variability: Intercept, %	6.78 (1.72, 11.84)	0.009	8.85 (4.81, 12.89)	<0.001
Magnitude-dependent variability: Slope	-0.06 (-0.14, 0.02)	0.160	-0.06 (-0.13, 0.00)	0.058
Error Slice: Figure 3Cii				
MAE, %	6.56 (5.48, 7.83)	-	7.55 (6.31, 8.91)	-
RMSE, %	8.17 (6.68, 9.82)	-	9.18 (7.76, 10.62)	-
R	0.19 (-0.01, 0.39)	-	0.32 (0.10, 0.50)	-
CCC	0.09 (-0.00, 0.20)	-	0.32 (0.10, 0.50)	-
R^2	0.03 (-0.12, 0.12)	-	-0.23 (-0.75, 0.09)	-
Proportional bias: Intercept, %	54.21 (47.07, 61.35)	<0.001	11.46 (-0.53, 23.44)	0.061
Proportional bias: Slope	-1.62 (-1.84, -1.41)	<0.001	-0.28 (-0.63, 0.07)	0.121
Magnitude-dependent variability: Intercept, %	2.98 (-0.33, 6.29)	0.078	4.98 (-1.98, 11.95)	0.161
Magnitude-dependent variability: Slope	0.01 (-0.09, 0.11)	0.882	-0.06 (-0.14, 0.27)	0.545
Error Slice: Supplementary Figure 2Bi				
MAE, %	9.19 (7.41, 11.19)	-	6.81 (5.65, 8.00)	-
RMSE, %	12.78 (9.91, 15.67)	-	8.85 (7.52, 10.14)	-
R	0.28 (-0.11, 0.58)	-	0.75 (0.55, 0.85)	-
CCC	0.08 (-0.03, 0.18)	-	0.68 (0.48, 0.79)	-
R^2	0.05 (-0.07, 0.15)	-	0.54 (0.26, 0.69)	-
Proportional bias: Intercept, %	103.07 (90.07, 116.08)	<0.001	24.79 (17.53, 32.04)	<0.001
Proportional bias: Slope	-1.78 (-2.00, -1.56)-	<0.001	-0.47 (-0.59, -0.35)	<0.001
Magnitude-dependent variability: Intercept, %	11.24 (4.71, 17.78)-	0.001	10.76 (5.94, 15.59	<0.001
Magnitude-dependent variability: Slope	--0.16 (-0.27, -0.04)	0.006	-0.09 (-0.17, -0.01)	0.030
Error Slice: Supplementary Figure 2Ci				
MAE, %	7.06 (6.13, 8.02)	-	7.37 (6.46, 8.30)	-
RMSE, %	8.78 (7.59, 9.97)	-	8.83 (7.73, 9.90)	-
R	0.77 (0.66, 0.85)	-	0.77 (0.66, 0.84)	-
CCC	0.70 (0.58, 0.79)	-	0.71 (0.58, 0.79)	-
R^2	0.58 (0.42, 0.68)	-	0.57 (0.41, 0.68)	-
Proportional bias: Intercept, %	27.01 (18.02, 36.00)	<0.001	24.71 (14.39, 35.03)	<0.001
Proportional bias: Slope	-0.47 (-0.62, -0.33)	<0.001	-0.45 (-0.61, -0.28)	<0.001
Magnitude-dependent variability: Intercept, %	12.60 (7.29, 17.91)	<0.001	12.97 (6.96, 18.98)	<0.001
Magnitude-dependent variability: Slope	-0.12 (-0.20, -0.03)	0.006	-0.12 (-0.22, -0.02)	0.014

Supplementary Table 7. LVEF estimation evaluation metrics on echocardiograms from PanEcho-Feats error slices

	EchoNet		PanEcho	
	**Value (95% CI)**	**P-value**	**Value (95% CI)**	**P-value**
Error Slice: Figure 3Di				
MAE, %	11.36 (8.29, 14.92)	-	6.60 (5.11, 8.29)	-
RMSE, %	15.48 (11.69, 19.15)	-	7.89 (6.03, 9.82)	-
R	0.14 (-0.07, 0.37)	-	0.47 (0.08, 0.72)	-
CCC	0.10 (-0.05, 0.26)	-	0.41 (0.06, 0.66)	-
R^2	-2.70 (-8.50, -0.90)	-	0.04 (-1.32, 0.48)	-
Proportional bias: Intercept, %	-12.58 (-29.42, 4.25)	0.143	17.22 (8.50, 25.95)	<0.001
Proportional bias: Slope	0.63 (0.02, 1.24	0.044	-0.47 (-0.74, -0.21)	<0.001
Magnitude-dependent variability: Intercept, %	-7.46 (-14.81, -0.10)	0.047	4.60 (-2.14, 11.34)	0.181
Magnitude-dependent variability: Slope	0.53 (0.30, 0.75)	<0.001	0.03 (-0.20, 0.26)	0.809
Error Slice: Figure 3Dii				
MAE, %	8.82 (6.84, 10.99)	-	8.45 (6.23, 10.64)	-
RMSE, %	10.48 (8.41, 12.73)	-	10.11 (7.98, 12.14)	-
R	0.26 (-0.05, 0.52)	-	0.21 (-0.05, 0.49)	-
CCC	0.11 (-0.02, 0.24)	-	0.07 (-0.02, 0.16)	-
R^2	-0.51 (-1.55, -0.08)	-	-0.41 (-1.36, -0.03)	-
Proportional bias: Intercept, %	79.79 (55.63, 103.95)	<0.001	99.79 (83.49, 116.09)	<0.001
Proportional bias: Slope	-1.30 (-1.66, -0.94)	<0.001	-1.58 (-1.82, -1.34)	<0.001
Magnitude-dependent variability: Intercept, %	13.50 (1.72, 25.28)	0.025	5.32 (-5.25, 15.89)	0.324
Magnitude-dependent variability: Slope	-0.14 (-0.31, 0.04)	0.120	-0.04 (-0.19, 0.12)	0.651
Error Slice: Supplementary Figure 2Di				
MAE, %	8.49 (6.40, 10.83)	-	8.33 (6.56, 10.02)	-
RMSE, %	11.10 (8.37, 13.83)	-	9.90 (8.11, 11.51)	-
R	0.21 (-0.13, 0.54)	-	0.37 (0.08, 0.59)	-
CCC	0.20 (-0.12, 0.50)	-	0.19 (0.05, 0.30)	-
R^2	-0.27 (-1.17, 0.23)	-	-0.01 (-0.41, 0.16)	-
Proportional bias: Intercept, %	19.38 (7.10, 31.65)	0.002	46.77 (37.12, 56.42)	<0.001
Proportional bias: Slope	-0.46 (-0.77, -0.15)	0.004	-1.33 (-1.56, -1.10)	<0.001
Magnitude-dependent variability: Intercept, %	9.17 (0.40, 17.93)	0.040	10.48 (5.60, 15.36)	<0.001
Magnitude-dependent variability: Slope	-0.02 (-0.23, 0.19)	0.840	-0.17 (-0.29, -0.05)	0.005
Error Slice: Supplementary Figure 2Dii				
MAE, %	8.14 (5.55, 10.89)	-	9.25 (6.83, 11.83)	-
RMSE, %	10.30 (7.07, 12.96)	-	10.83 (8.36, 13.17)	-
R	0.52 (0.23, 0.79)	-	0.49 (0.09, 0.77)	-
CCC	0.38 (0.14, 0.62)	-	0.23 (0.04, 0.33)	-
R^2	-0.22 (-0.73, 0.49)	-	0.14 (-0.75, 0.28)	-
Proportional bias: Intercept, %	54.51 (26.84, 82.19)	<0.001	83.94 (72.58, 95.30)	<0.001
Proportional bias: Slope	-0.94 (-1.37, -0.50)	<0.001	-1.42 (-1.60, -1.24)	<0.001
Magnitude-dependent variability: Intercept, %	14.16 (4.29, 24.03)	0.005	5.17 (-6.32, 16.67)	0.378
Magnitude-dependent variability: Slope	-0.14 (-0.30, 0.02)	0.083	-0.03 (-0.21, 0.16)	0.765

Supplementary Table 8. Patient- and study-level characteristics of studies inside versus outside LV segmentator error slices

Characteristic	In-slice	Out-slice	Effect (95% CI)	P-value
Error Slice: Figure 3Bi				
Age, years—mean (SD)	69.9 (14.1)	71.6 (13.2)	-1.63 (-6.49, 3.23)	0.511
Sex, Male vs. Female—No. (%)	13 (39.4)	618 (47.8)	0.71 (0.35, 1.44)	0.340
Race, White vs. Non-White—No. (%)	11 (64.7)	491 (70.4)	0.77 (0.28, 2.11)	0.610
Obesity, BMI ≥30 kg/m^2—No. (%)	7 (21.2)	404 (30.6)	0.61 (0.26, 1.42)	0.251
Ultrasound Device: Vivid E95 vs. others—No. (%)	14 (42.4)	629 (47.7)	0.81 (0.40, 1.63)	0.554
Ultrasound Device: Vivid S70 vs. others—No. (%)	18 (54.5)	603 (45.7)	1.43 (0.71, 2.86)	0.316
Ultrasound Device: Vivid E90 vs. others—No. (%)	1 (3.0)	88 (6.7)	0.44 (0.06, 3.24)	0.419
Reference biplane LVEF, %—mean (SD)	59.3 (14.8)	58.4 (13.2)	0.93 (-4.17, 6.03)	0.720
Error Slice: Figure 3Bii				
Age, years—mean (SD)	71.6 (14.2)	71.5 (13.0)	0.09 (-2.21, 2.40)	0.938
Sex, Male vs. Female—No. (%)	80 (48.8)	551 (47.5)	1.05 (0.76, 1.46)	0.751
Race, White vs. Non-White—No. (%)	70 (73.7)	432 (69.8)	1.21 (0.74, 1.98)	0.440
Obesity, BMI ≥30 kg/m^2—No. (%)	56 (33.7)	355 (29.9)	1.19 (0.84, 1.68)	0.316
Ultrasound Device: Vivid E95 vs. others—No. (%)	78 (47.0)	565 (47.6)	0.98 (0.70, 1.35)	0.883
Ultrasound Device: Vivid S70 vs. others—No. (%)	82 (49.4)	539 (45.4)	1.17 (0.85, 1.62)	0.335
Ultrasound Device: Vivid E90 vs. others—No. (%)	6 (3.6)	83 (7.0)	0.50 (0.21, 1.16)	0.107
Reference biplane LVEF, %—mean (SD)	60.4 (11.9)	58.2 (13.4)	2.21 (0.25, 4.18)	0.027
Error Slice: Supplementary Figure 2Ai				
Age, years—mean (SD)	73.8 (12.6)	71.5 (13.2)	2.31 (-2.41, 7.04)	0.337
Sex, Male vs. Female—No. (%)	15 (53.6)	616 (47.5)	1.28 (0.60, 2.70)	0.525
Race, White vs. Non-White—No. (%)	12 (66.7)	490 (70.4)	0.84 (0.31, 2.27)	0.733
Obesity, BMI ≥30 kg/m^2—No. (%)	10 (35.7)	401 (30.3)	1.28 (0.59, 2.80)	0.536
Ultrasound Device: Vivid E95 vs. others—No. (%)	12 (42.9)	631 (47.6)	0.82 (0.39, 1.76)	0.618
Ultrasound Device: Vivid S70 vs. others—No. (%)	15 (53.6)	606 (45.7)	1.37 (0.65, 2.90)	0.412

Ultrasound Device: Vivid E90 vs. others—No. (%)	1 (3.6)	88 (6.6)	0.52 (0.07, 3.88)	0.524
Reference biplane LVEF, %—mean (SD)	52.3 (20.3)	58.6 (13.0)	-6.27 (-13.82, 1.29)	0.104
Error Slice: Supplementary Figure 2Aii				
Age, years—mean (SD)	72.1 (12.3)	-71.5 (13.3)	0.66 (-2.00, 3.31)	0.629
Sex, Male vs. Female—No. (%)	37 (41.6)	594 (48.1)	0.77 (0.50, 1.19)	0.238
Race, White vs. Non-White—No. (%)	30 (65.2)	472/668 (70.7)	0.78 (0.41, 1.46)	0.436
Obesity, BMI ≥30 kg/m^2—No. (%)	43 (47.8)	368/1263 (29.1)	2.23 (1.45, 3.43)	<0.001
Ultrasound Device: Vivid E95 vs. others—No. (%)	45 (50.0)	598 (47.3)	1.11 (0.72, 1.71)	0.627
Ultrasound Device: Vivid S70 vs. others—No. (%)	39 (43.3)	582 (46.1)	0.89 (0.58, 1.38)	0.614
Ultrasound Device: Vivid E90 vs. others—No. (%)	6 (6.7)	83 (6.6)	1.02 (0.43, 2.40)	0.972
Reference biplane LVEF, %—mean (SD)	56.0 (15.6)	58.6 (13.0)	-2.55 (-5.85, 0.75)	0.130

Supplementary Table 9. Patient- and study-level characteristics of studies inside versus outside EchoNet-Feats error slices

Characteristic	In-slice	Out-slice	Effect (95% CI)	P-value
Error Slice: Figure 3Ci				
Age, years—mean (SD)	72.3 (12.9)	71.5 (13.2)	0.77 (-2.92, 4.47)	0.681
Sex, Male vs. Female—No. (%)	26 (53.1)	605 (47.4)	1.25 (0.71, 2.22)	0.439
Race, White vs. Non-White—No. (%)	20 (71.4)	482 (70.3)	1.06 (0.46, 2.44)	0.895
Obesity, BMI ≥30 kg/m^2—No. (%)	22 (44.0)	389 (29.9)	1.85 (1.04, 3.27)	0.035
Ultrasound Device: Vivid E95 vs. others—No. (%)	16 (32.0)	627 (48.1)	0.51 (0.28, 0.93)	0.028
Ultrasound Device: Vivid S70 vs. others—No. (%)	27 (54.0)	594 (45.6)	1.40 (0.79, 2.47)	0.244
Ultrasound Device: Vivid E90 vs. others—No. (%)	7 (14.0)	82 (6.3)	2.42 (1.06, 5.56)	0.037
Reference biplane LVEF, %—mean (SD)	54.9 (17.1)	58.6 (13.0)	-3.68 (-8.49, 1.13)	0.134
Error Slice: Figure 3Cii				
Age, years—mean (SD)	74.0 (12.2)	71.4 (13.2)	2.62 (-0.63, 5.87)	0.114
Sex, Male vs. Female—No. (%)	36 (63.2)	595 (46.9)	1.94 (1.12, 3.36)	0.018
Race, White vs. Non-White—No. (%)	21 (55.3)	481 (71.2)	0.50 (0.26, 0.97)	0.041
Obesity, BMI ≥30 kg/m^2—No. (%)	17 (29.3)	394 (30.4)	0.95 (0.53, 1.69)	0.857
Ultrasound Device: Vivid E95 vs. others—No. (%)	17 (29.3)	626 (48.3)	0.44 (0.25, 0.79)	0.006
Ultrasound Device: Vivid S70 vs. others—No. (%)	38 (65.5)	583 (45.0)	2.32 (1.34, 4.03)	0.003
Ultrasound Device: Vivid E90 vs. others—No. (%)	3 (5.2)	86 (6.6)	0.77 (0.23, 2.50)	0.660
Reference biplane LVEF, %—mean (SD)	33.1 (8.8)	59.6 (12.2)	-26.49 (-28.86, -24.12)	<0.001
Error Slice: Supplementary Figure 2Bi				
Age, years—mean (SD)	74.2 (13.0)	71.3 (13.2)	2.90 (0.04, 5.76)	0.047
Sex, Male vs. Female—No. (%)	44 (51.8)	587 (47.3)	1.19 (0.77, 1.85)	0.430
Race, White vs. Non-White—No. (%)	34 (68.0)	468 (70.5)	0.89 (0.48, 1.65)	0.712
Obesity, BMI ≥30 kg/m^2—No. (%)	25 (29.4)	386 (30.4)	0.95 (0.59, 1.54)	0.842
Ultrasound Device: Vivid E95 vs. others—No. (%)	43 (50.6)	600 (47.3)	1.14 (0.73, 1.77)	0.559
Ultrasound Device: Vivid S70 vs. others—No. (%)	40 (47.1)	581 (45.8)	1.05 (0.68, 1.63)	0.825

Ultrasound Device: Vivid E90 vs. others—No. (%)	2 (2.4)	87 (6.9)	0.33 (0.08, 1.35)	0.123
Reference biplane LVEF, %—mean (SD)	56.2 (13.0)	58.6 (13.2)	-2.40 (-5.26, 0.46)	0.100
Error Slice: Supplementary Figure 2Bii				
Age, years—mean (SD)	70.4 (13.5)	71.6 (13.2)	-1.22 (-3.78, 1.34)	0.351
Sex, Male vs. Female—No. (%)	56 (47.9)	575 (47.6)	1.01 (0.69, 1.48)	0.957
Race, White vs. Non-White—No. (%)	46 (67.6)	456 (70.6)	0.87 (0.51, 1.49)	0.614
Obesity, BMI ≥30 kg/m^2—No. (%)	33 (28.0)	378 (30.6)	0.88 (0.58, 1.34)	0.552
Ultrasound Device: Vivid E95 vs. others—No. (%)	62 (52.5)	581 (47.0)	1.25 (0.85, 1.82)	0.254
Ultrasound Device: Vivid S70 vs. others—No. (%)	52 (44.1)	569 (46.1)	0.92 (0.63, 1.35)	0.677
Ultrasound Device: Vivid E90 vs. others—No. (%)	4 (3.4)	85 (6.9)	0.47 (0.17, 1.32)	0.153
Reference biplane LVEF, %—mean (SD)	58.9 (13.4)	58.4 (13.2)	0.56 (-1.97, 3.08)	0.666

Supplementary Table 10. Patient- and study-level characteristics of studies inside versus outside PanEcho-Feats error slices

Characteristic	In-slice	Out-slice	Effect (95% CI)	P-value
Error Slice: Figure 3Di				
Age, years—mean (SD)	81.1 (10.0)	71.3 (13.2)	9.85 (6.51, 13.20)	<0.001
Sex, Male vs. Female—No. (%)	16 (44.4)	615 (47.7)	0.88 (0.45, 1.71)	0.699
Race, White vs. Non-White—No. (%)	25 (78.1)	477 (69.9)	1.53 (0.65, 3.61)	0.326
Obesity, BMI ≥30 kg/m^2—No. (%)	3 (8.3)	408 (31.0)	0.20 (0.06, 0.66)	0.008
Ultrasound Device: Vivid E95 vs. others—No. (%)	8 (22.2)	635 (48.2)	0.31 (0.14, 0.68)	0.004
Ultrasound Device: Vivid S70 vs. others—No. (%)	27 (75.0)	594 (45.1)	3.65 (1.70, 7.83)	0.001
Ultrasound Device: Vivid E90 vs. others—No. (%)	1 (2.8)	88 (6.7)	0.40 (0.05, 2.95)	0.368
Reference biplane LVEF, %—mean (SD)	28.7 (7.8)	59.2 (12.4)	-30.51 (-33.14, -27.88)	<0.001
Error Slice: Figure 3Dii				
Age, years—mean (SD)	80.9 (11.0)	71.3 (13.2)	9.57 (5.70, 13.44)	<0.001
Sex, Male vs. Female—No. (%)	5 (15.6)	626 (48.4)	0.20 (0.08, 0.52)	0.001
Race, White vs. Non-White—No. (%)	13 (86.7)	489 (70.0)	2.79 (0.62, 12.50)	0.180
Obesity, BMI ≥30 kg/m^2—No. (%)	13 (40.6)	398 (30.1)	1.59 (0.78, 3.25)	0.206
Ultrasound Device: Vivid E95 vs. others—No. (%)	17 (53.1)	626 (47.4)	1.26 (0.62, 2.54)	0.522
Ultrasound Device: Vivid S70 vs. others—No. (%)	12 (37.5)	609 (46.1)	0.70 (0.34, 1.45)	0.337
Ultrasound Device: Vivid E90 vs. others—No. (%)	3 (9.4)	86 (6.5)	1.49 (0.44, 4.98)	0.521
Reference biplane LVEF, %—mean (SD)	69.2 (9.2)	58.2 (13.2)	11.09 (7.82, 14.36)	<0.001
Error Slice: Supplementary Figure 2Di				
Age, years—mean (SD)	67.6 (12.6)	71.6 (13.2)	-4.04 (-8.21, 0.13)	0.058
Sex, Male vs. Female—No. (%)	20 (55.6)	611 (47.4)	1.39 (0.71, 2.70)	0.336
Race, White vs. Non-White—No. (%)	11 (50.0)	491 (71.0)	0.41 (0.17, 0.96)	0.040
Obesity, BMI ≥30 kg/m^2—No. (%)	8 (22.2)	403 (30.6)	0.65 (0.29, 1.43)	0.285
Ultrasound Device: Vivid E95 vs. others—No. (%)	15 (41.7)	628 (47.7)	0.78 (0.40, 1.53)	0.477
Ultrasound Device: Vivid S70 vs. others—No. (%)	19 (52.8)	602 (45.7)	1.33 (0.68, 2.58)	0.403

Ultrasound Device: Vivid E90 vs. others—No. (%)	2 (5.6)	87 (6.6)	0.83 (0.20, 3.52)	0.802
Reference biplane LVEF, %—mean (SD)	39.1 (10.2)	59.0 (12.9)	-19.90 (-23.31, -16.49)	<0.001
Error Slice: Supplementary Figure 2Dii				
Age, years—mean (SD)	68.8 (11.5)	71.6 (13.2)	-2.75 (-7.72, 2.23)	0.279
Sex, Male vs. Female—No. (%)	15 (71.4)	616 (47.2)	2.79 (1.08, 7.25)	0.035
Race, White vs. Non-White—No. (%)	10 (58.8)	492 (70.6)	0.60 (0.22, 1.59)	0.300
Obesity, BMI ≥30 kg/m^2—No. (%)	12 (57.1)	399 (30.0)	3.12 (1.30, 7.46)	0.011
Ultrasound Device: Vivid E95 vs. others—No. (%)	8 (38.1)	635 (47.7)	0.68 (0.28, 1.64)	0.386
Ultrasound Device: Vivid S70 vs. others—No. (%)	13 (61.9)	608 (45.6)	1.94 (0.80, 4.70)	0.145
Ultrasound Device: Vivid E90 vs. others—No. (%)	0 (0.0)	89 (6.7)	-	-
Reference biplane LVEF, %—mean (SD)	61.9 (11.9)	58.4 (13.2)	3.49 (-1.67, 8.64)	0.185

Supplementary Table 11. Differences between selective estimation AURC and no-selection MAE

	EchoNet	PanEcho
View Classifier	-0.45 (-0.86, -0.02)	-0.35 (-0.78, 0.11)
LV Segmentator	0.03 (-0.56, 0.64)	0.17 (-0.29, 0.66)
EchoNet-Feats	-1.08 (-1.52, -0.63)	-0.63 (-1.09, -0.17)
PanEcho-Feats	-0.44 (-0.95, 0.04)	-0.33 (-0.74, 0.07)

Differences between selective estimation AURC and no-selection MAE for each combination of LVEF estimation model and deep feature representation. Point values correspond to the differences computed across the entire held-out test set, and sets correspond to 95% confidence intervals that were computed using a paired patient-level bootstrap on the held-out test set, with patients resampled with replacement over 10,000 iterations. Differences are significant when their 95% bootstrap CI excludes zero, and negative differences imply that selective estimation improved performance relative to standard AI deployment.
Abbreviations: AURC = area under the risk-coverage curve; LV = left ventricle; MAE = mean absolute error

Supplementary Table 12. TRIPOD+AI Checklist

Section / Topic	Item	Development / evaluation[1]	Checklist item	Reported on page / section
**TITLE**				
Title	1	D;E	Identify the study as developing or evaluating the performance of a multivariable prediction model, the target population, and the outcome to be predicted.	Title Page (Pg. 1)
**ABSTRACT**				
Abstract	2	D;E	See TRIPOD+AI for Abstracts checklist.	Abstract (Pg. 2-3)
**INTRODUCTION**				
Background	3a	D;E	Explain the healthcare context (including whether diagnostic or prognostic) and rationale for developing or evaluating the prediction model, including references to existing models.	Introduction (Pg. 5-6)
Background	3b	D;E	Describe the target population and the intended purpose of the prediction model in the context of the care pathway, including its intended users (e.g., healthcare professionals, patients, public).	Introduction (Pg. 5)
Background	3c	D;E	Describe any known health inequalities between sociodemographic groups.	Introduction (Pg. 5)
Objectives	4	D;E	Specify the study objectives, including whether the study describes the development or validation of a prediction model (or both).	Introduction (Pg. 6)
**METHODS**				
Data	5a	D;E	Describe the sources of data separately for the development and evaluation datasets (e.g., randomised trial, cohort, routine care or registry data), the rationale for using these data, and representativeness of the data.	Methods, Data (Pg. 7); Supplementary Methods (Supp. Pg. 3)
Data	5b	D;E	Specify the dates of the collected participant data, including start and end of participant accrual; and, if applicable, end of follow-up.	Methods, Data (Pg. 7); Supplementary Methods (Supp. Pg. 3)
Participants	6a	D;E	Specify key elements of the study setting (e.g., primary care, secondary care, general population) including the number and location of centres.	Methods, Data (Pg. 7); Supplementary Methods (Supp. Pg. 3)
Participants	6b	D;E	Describe the eligibility criteria for study participants.	Methods, Data (Pg. 7); Supplementary Methods (Supp. Pg. 3) Supplementary Figure 1

Section / Topic	Item	Development / evaluation[1]	Checklist item	Reported on page / section
Participants	6c	D;E	Give details of any treatments received, and how they were handled during model development or evaluation, if relevant.	N/A
Data preparation	7	D;E	Describe any data pre-processing and quality checking, including whether this was similar across relevant sociodemographic groups.	Methods, Data (Pg. 7); Supplementary Methods (Supp. Pg. 3) Supplementary Figure 1
Outcome	8a	D;E	Clearly define the outcome that is being predicted and the time horizon, including how and when assessed, the rationale for choosing this outcome, and whether the method of outcome assessment is consistent across sociodemographic groups.	Methods (Pg. 7-12)
Outcome	8b	D;E	If outcome assessment requires subjective interpretation, describe the qualifications and demographic characteristics of the outcome assessors.	Methods (Pg. 7-12)
Outcome	8c	D;E	Report any actions to blind assessment of the outcome to be predicted.	Methods (Pg. 7-12)
Predictors	9a	D	Describe the choice of initial predictors (e.g., literature, previous models, all available predictors) and any pre-selection of predictors before model building.	Methods (Pg. 7-12)
Predictors	9b	D;E	Clearly define all predictors, including how and when they were measured (and any actions to blind assessment of predictors for the outcome and other predictors).	Methods (Pg. 7-12)
Predictors	9c	D;E	If predictor measurement requires subjective interpretation, describe the qualifications and demographic characteristics of the predictor assessors.	Methods (Pg. 7-12)
Sample size	10	D;E	Explain how the study size was arrived at (separately for development and evaluation), and justify that the study size was sufficient to answer the research question. Include details of any sample size calculation.	Methods, Data (Pg 7), Selective Estimation (Pg. 10); Supplementary Methods (Supp. Pg. 3); Supplementary Figure 1
Missing data	11	D;E	Describe how missing data were handled. Provide reasons for omitting any data.	Supplementary Methods (Pg. 3); Supplementary Figure 1

Section / Topic	Item	Development / evaluation[1]	Checklist item	Reported on page / section
Analytical methods	12a	D	Describe how the data were used (e.g., for development and evaluation of model performance) in the analysis, including whether the data were partitioned, considering any sample size requirements.	Methods, Statistical Analysis (Pg. 10-12)
Analytical methods	12b	D	Depending on the type of model, describe how predictors were handled in the analyses (functional form, rescaling, transformation, or any standardisation).	Methods (Pg. 7-12)
Analytical methods	12c	D	Specify the type of model, rationale, all model-building steps, including any hyperparameter tuning, and method for internal validation.	Methods (Pg. 7-12)
Analytical methods	12d	D;E	Describe if and how any heterogeneity in estimates of model parameter values and model performance was handled and quantified across clusters (e.g., hospitals, countries). See TRIPOD-Cluster for additional considerations.	Methods, Statistical Analysis (Pg. 10-12)
Analytical methods	12e	D;E	Specify all measures and plots used (and their rationale) to evaluate model performance (e.g., discrimination, calibration, clinical utility) and, if relevant, to compare multiple models.	Methods, Statistical Analysis (Pg. 10-12)
Analytical methods	12f	E	Describe any model updating (e.g., recalibration) arising from the model evaluation, either overall or for particular sociodemographic groups or settings.	N/A
Analytical methods	12g	E	For model evaluation, describe how the model predictions were calculated (e.g., formula, code, object, application programming interface).	Methods, Statistical Analysis (Pg. 10-12)
Class imbalance	13	D;E	If class imbalance methods were used, state why and how this was done, and any subsequent methods to recalibrate the model or the model predictions.	N/A
Fairness	14	D;E	Describe any approaches that were used to address model fairness and their rationale.	N/A
Model output	15	D	Specify the output of the prediction model (e.g., probabilities, classification). Provide details and rationale for any classification and how the thresholds were identified.	Methods (Pg. 7-12)
Training vs. evaluation	16	D;E	Identify any differences between the development and evaluation data in healthcare setting, eligibility criteria, outcome, and predictors.	Methods, Selective Estimation (Pg. 9-10); Supplementary Figure 1
Ethical approval	17	D;E	Name the institutional research board or ethics committee that approved the study and describe	Methods, Data Ethics and

Section / Topic	Item	Development / evaluation[1]	Checklist item	Reported on page / section
			the participant-informed consent or the ethics committee waiver of informed consent.	Software Availability (Pg 13)
**OPEN SCIENCE**				
Funding	18a	D;E	Give the source of funding and the role of the funders for the present study.	Title Page (Pg 1)
Conflicts of interest	18b	D;E	Declare any conflicts of interest and financial disclosures for all authors.	Title Page (Pg 1)
Protocol	18c	D;E	Indicate where the study protocol can be accessed or state that a protocol was not prepared.	N/A
Registration	18d	D;E	Provide registration information for the study, including register name and registration number, or state that the study was not registered.	N/A
Data sharing	18e	D;E	Provide details of the availability of the study data.	Methods, Data Ethics and Software Availability (Pg 14-15)
Code sharing	18f	D;E	Provide details of the availability of the analytical code.	Methods, Data Ethics and Software Availability (Pg 14-15)
**PATIENT & PUBLIC INVOLVEMENT**				
Patient & Public Involvement	19	D;E	Provide details of any patient and public involvement during the design, conduct, reporting, interpretation, or dissemination of the study or state no involvement.	N/A
**RESULTS**				
Participants	20a	D;E	Describe the flow of participants through the study, including the number of participants with and without the outcome and, if applicable, a summary of the follow-up time. A diagram may be helpful.	Methods, Data (Pg. 6-7); Supplementary Methods (Pg. 2); Supplementary Figure 1.
Participants	20b	D;E	Report the characteristics overall and, where applicable, for each data source or setting, including the key dates, key predictors (including demographics), treatments received, sample size, number of outcome events, follow-up time, and amount of missing data. A table may be helpful. Report any differences across key demographic groups.	Supplementary Table 1
Participants	20c	E	For model evaluation, show a comparison with the development data of the distribution of important	Supplementary Table 1

Section / Topic	Item	Development / evaluation[1]	Checklist item	Reported on page / section
			predictors (demographics, predictors, and outcome).	
Model development	21	D;E	Specify the number of participants and outcome events in each analysis (e.g., for model development, hyperparameter tuning, model evaluation).	Supplementary Table 1
Model specification	22	D	Provide details of the full prediction model (e.g., formula, code, object, application programming interface) to allow predictions in new individuals and to enable third-party evaluation and implementation, including any restrictions to access or re-use (e.g., freely available, proprietary).	Methods (Pg. 6-13); Supplementary Methods (Pg. 2)
Model performance	23a	D;E	Report model performance estimates with confidence intervals, including for any key subgroups (e.g., sociodemographic). Consider plots to aid presentation.	Tables 1-5
Model performance	23b	D;E	If examined, report results of any heterogeneity in model performance across clusters. See TRIPOD-Cluster for additional details.	N/A
Model updating	24	E	Report the results from any model updating, including the updated model and subsequent performance.	N/A
**DISCUSSION**				
Interpretation	25	D;E	Give an overall interpretation of the main results, including issues of fairness in the context of the objectives and previous studies.	Discussion (Pg. 16-19)
Limitations	26	D;E	Discuss any limitations of the study (such as a non-representative sample, sample size, overfitting, missing data) and their effects on any biases, statistical uncertainty, and generalizability.	Limitations (Pg. 19-20)
Usability of the model in the context of current care	27a	D	Describe how poor quality or unavailable input data (e.g., predictor values) should be assessed and handled when implementing the prediction model.	Discussion (Pg. 17-19)
Usability of the model in the context of current care	27b	D	Specify whether users will be required to interact in the handling of the input data or use of the model, and what level of expertise is required of users.	Clinical Perspectives (Pg. 21)
Usability of the model in the context of current care	27c	D;E	Discuss any next steps for future research, with a specific view to applicability and generalizability of the model.	Limitations (Pg. 19-20); Clinical Perspectives (Pg. 21)

From: Collins GS, Moons KGM, Dhiman P, et al. BMJ 2024;385:e078378. doi:10.1136/bmj-2023-078378 (checklist version 11-January-2024).
[1]D = items relevant only to the development of a prediction model; E = items relating solely to the evaluation of a prediction model; D;E = items applicable to both.
